# Scalable synthetic peptide hydrogel enables self-organized luminal cavity architecture within hiPSC 3D colonies supporting functional hepatocyte differentiation

**DOI:** 10.1016/j.bioactmat.2026.03.029

**Published:** 2026-04-01

**Authors:** Quan Li, Guangyan Qi, Ling Cai, Abhishek P. Shrestha, Sergio Duarte, Yu Shrike Zhang, Ali Zarrinpar, Tawfik Aboellail, Xuming Liu, Jianfa Bai, Md Sharifur Rahman, Bruno Hagenbuch, Ming-Shun Chen, Anthony Atala, Xiuzhi Susan Sun

**Affiliations:** a Department of Biological and Agricultural Engineering, Kansas State University, Manhattan, KS, USA; b Institute of Regenerative Medicine, Wake Forest University School of Medicine, Winston Salem, NC, USA; c Division of Engineering in Medicine, Department of Medicine, Brigham and Women's Hospital, Harvard Medical School, Cambridge, MA, USA; d Department of Surgery, University of Florida, Gainesville, FL, USA; e Kansas State Veterinary Diagnostic Medicine and Pathology, College of Veterinary Medicine, Kansas State University, Manhattan, KS, USA; f USDA-ARE and Department of Entomology, Kansas State University, Manhattan, KS, USA; g Kansas State Veterinary Diagnostic Laboratory, College of Veterinary Medicine, Kansas State University, Manhattan, KS, USA; h School of Medicine, The University of Kansas Medical Center, Kansas City, KS, USA

**Keywords:** Synthetic peptide hydrogel, Pluripotent stem cells, Functional hepatocytes, 3D organoids, Bioprinting

## Abstract

Human induced pluripotent stem cells (hiPSCs) possess broad differentiation potential; however, efficient maturation into functional hepatocytes remains challenging, in part because conventional differentiation strategies rely on 2D culture or partially defined 3D systems that fail to recapitulate key developmental microenvironmental cues. Although 3D culture has been shown to promote hepatic specification and maturation, a fully defined platform that supports the entire differentiation process from hiPSCs in 3D has not been established. Here, we report a scalable synthetic peptide hydrogel (PepGel) platform that enables hiPSCs to self-organize into long-term, proliferative luminal cavity (LC) architectures within 3D colonies (LC-hiPSCs). These LC-hiPSCs exhibited markedly enhanced differentiation efficiency compared to hiPSC aggregates generated via scaffold-free suspension methods. Using a shear-thinning, self-healing hydrogel configuration optimized for suspension culture, we produced large quantities of LC-hiPSCs and directed their differentiation into PepGel-derived hiPSC-derived hepatocyte-like cells (PG-hiHs). PG-hiHs formed polarized, multi-luminal organoid-like structures and exhibited robust expression of hepatocyte-specific genes and proteins, high cytochrome P450 activity, and in vitro metabolic functions comparable to primary human hepatocytes (PHHs). Following live shipment and transplantation into immunocompromised mouse livers, PG-hiHs engrafted efficiently and maintained human-specific albumin and alpha-1 antitrypsin secretion at PHH-equivalent levels. Furthermore, both LC-hiPSCs and PG-hiHs retained viability, structural organization, and phenotype in complex 3D bioprinted constructs for at least 14 days. Together, this work establishes a fully defined, reproducible, and scalable 3D platform that supports the entire differentiation process, overcoming limitations in hiPSC-derived hepatic maturation and enables physiologically relevant hepatocyte-like tissues for disease modeling, regenerative medicine, and biofabrication.

## Highlights

• Fully defined, tunable synthetic peptide hydrogel directs hiPSC self-organization into luminal-cavity (LC) 3D architecture.
• LC-hiPSCs enable efficient, end-to-end 3D differentiation into functional hepatocyte-like cells or organoids (PG-hiHs).
• PG-hiHs exhibit cytochrome P450 activity and metabolic functions comparable to primary human hepatocytes.
• LC-hiPSCs and PG-hiHs retain viability, phenotype, function after live shipping, in vivo engraftment, and 3D bioprinting.
• The platform supports scalable 3D biomanufacturing of hepatic cells or organoids in a single synthetic hydrogel system.

## Graphical abstract

**Figure:**
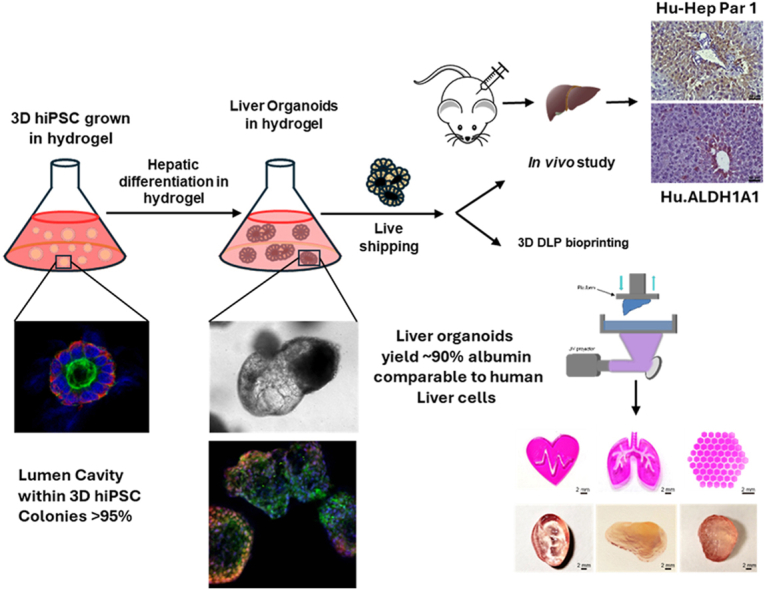

## 1 Introduction

Pluripotent stem cells (PSCs) including human induced PSCs (hiPSCs) represent a ground state of pluripotency, characterized by unique developmental potentials and distinct molecular profiles. Importantly, PSCs undergo early lineage specification in vivo within spatially organized, polarized epithelial structures rather than as disordered cell aggregates [1,2]. Understanding PSC behavior, growth, and differentiation potential within defined and controllable synthetic 3D microenvironments is therefore critical for advancing regenerative medicine and tissue engineering applications. Recent studies have explored the intrinsic ability of human PSCs to self-organize into luminal cavity (LC)-containing epithelial architectures, which resemble early epiblast morphogenesis. For instance, research has demonstrated that dissociated hESCs are intrinsically programmed to form lumens on 2D Geltrex (Matrigel based gel), creating two-cell cysts with a shared apical domain within 20 h of plating [1]. These cysts eventually collapse to form monolayers after five days, while maintaining the expression of pluripotency markers throughout this process [1]. Geltrex overlay methods promoted LC structure formation of hESC when ROCK inhibitor Y-27632 (ROCKi) was removed from culture [2], which was used as a model to mimic early developmental processes. These LC structures were shown to establish apical–basal polarity and spatially resolved signaling domains, key regulators of lineage commitment [[1], [2], [3], [4]]. However, PSC-derived luminal structures generated under these conditions are short-lived, lack proliferative capacity, and rapidly collapse, limiting their utility for long-term expansion or differentiation.

PSCs have been widely used to generate human organoids for drug screening and limited preclinical research; however, most PSC-derived organoids remain developmentally immature [5]. In these approaches, PSCs are typically expanded in 2D before aggregation into spheroids using ultra-low attachment plates or scaffold-free suspension cultures [5]. As a result, these spheroids arise through stochastic cell aggregation and lack intrinsic LC architecture, contributing to impaired epithelial polarity and limited functional maturation. In contrast, functional organoids derived from primary human cells replicate cellular heterogeneity and serve as valuable models for studying development and diseases [6], but they lack self-renewal capability and depend on fresh surgical tissues, severely limiting scalability and translational applicability.

We hypothesized that preserving and expanding the intrinsic LC architecture of hiPSCs would establish a developmentally relevant niche that enhances differentiation efficiency and functional maturation. Our initial studies demonstrated that hiPSC 3D colonies containing stable LC (LC-hiPSCs) exhibited remarkably enhanced differentiation potential towards endoderm lineage, and therefore, in this study, hepatocytes were selected as a representative endodermal lineage due to the availability of established maturation benchmarks. Notably, despite extensive efforts, engineered hepatocytes with functional equivalence to primary human hepatocytes (PHHs) remain an unmet need [[7], [8], [9], [10], [11], [12], [13], [14], [15], [16]]. One major barrier to clinical translation is that most existing growth microenvironments are lack of well-defined LC formation in 3D conditions [[17], [18], [19], [20], [21], [22]], leading to high variability and inconsistent organoid phenotypes. Although LC formation is an intrinsic property of PSCs, it cannot be stably maintained in 2D culture, animal-derived extracellular matrices (ECMs) such as Matrigel or Geltrex [1,2], or scaffold-free spheroid systems [18,[23], [24], [25], [26], [27]]. Moreover, reliance solely on cell-intrinsic self-organization (e.g., Geltrex overlay methods [2]) provides limited experimental control over morphogenesis, lineage specification, and scalability [6]. We initially tested Matrigel and vitronectin as potential hydrogel candidates for comparison; however, neither material was able to support stable LC-hiPSC formation at the outset. Therefore, we selected the scaffold-free suspension system, which is commonly used in hepatic differentiation, as the appropriate comparison group.

In this study, we employed a fully defined synthetic peptide hydrogel (PepGel) platform [28], specifically designed to support long-term maintenance, expansion, and differentiation of LC-hiPSCs. Unlike natural ECM-based matrices [[19], [20], [21], [22]], PepGel provides a chemically defined, tunable, and reproducible 3D environment that enables external control over mechanical and transport properties. LC-hiPSCs were expanded and subsequently differentiated into hepatocyte 3D colonies (organoids) using the same PepGel formulation, enabling a continuous, end-to-end 3D differentiation process. Beyond providing a nanofibrous microenvironment, PepGel enables efficient recovery of intact LC-hiPSC colonies via centrifugation, facilitating scalable bioprocessing. This hydrogel, constructed from human muscle and spider silk protein domains, representing synthetic ECM, is built on the h9e tri-block synthetic peptides [[29], [30], [31], [32]] and has proven to be cytocompatible and nonimmunogenic [31,[33], [34], [35], [36], [37], [38], [39], [40], [41], [42], [43]], exhibiting high performance in 3D hiPSC cultures [28]. However, whether this platform could support efficient differentiation of LC-hiPSCs into hepatocyte-like cells with PHH-relevant physiological functions had not been previously investigated.

We hypothesized that integrating intrinsic LC-driven self-organization of hiPSCs with a defined synthetic peptide hydrogel would enable generation of self-organized hepatocyte 3D colonies exhibiting physiological functions comparable to PHHs. Such a platform would support not only in vivo translational applications but also advanced tissue engineering approaches, including 3D bioprinting. To evaluate differentiation efficiency and scalability, we compared three differentiation routes: embedded matrix, suspension matrix, and scaffold-free suspension culture, across varying gel strengths (Fig. 2e).

**Fig. 1:**
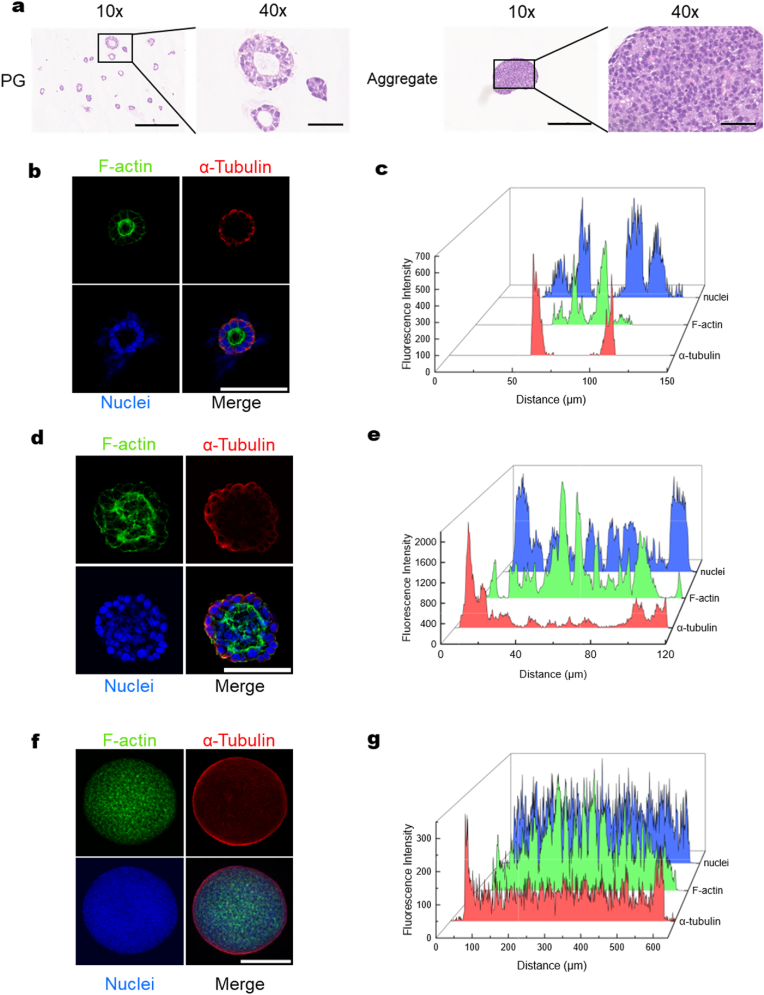
**Morphology of hiPSCs differs when they are generated in different 3D systems. a,** H&E staining of 3D LC-hiPSC colonies grown in PG-embedded or aggregated spheroids formed in a nonadherent suspension (aggregate) at low (10x) and high (40x) magnifications. The scale bars are 600 μm for 10x and 120 μm for 40x. **b,** LC-hiPSC 3D colonies grown in PG hydrogel were stained for the cytoskeleton components F-actin (green), α-tubulin (red) and nuclei (blue). Scale bar, 200 μm. **c,** Fluorescence intensity for F-actin, α-tubulin and nuclei from Fig. 1b was quantified and shown as intensity along a line from the left to the right edge across the center of the LC-hiPSCs colonies. **d,** hiPSC 3D colonies grown in PG-suspension hydrogel were stained for the cytoskeleton components F-actin (green), α-tubulin (red) and nuclei (blue). Scale bar, 200 μm. **e,** Fluorescence intensity for F-actin, α-tubulin and nuclei from Fig. 1d was quantified and shown as intensity along a line from the left edge to the right edge across the center of the spheroid. **f,** hiPSC Aggregates formed from single cells in a nonadherent suspension were stained for the cytoskeleton component F-actin (green), α-tubulin (red) and nuclei (blue). Scale bar, 600 μm **g,** Fluorescence intensity for F-actin, α-tubulin and nuclei from Fig. 1d was quantified and shown as intensity along a line from the left to the right edge across the center of the aggregated spheroid.

**Fig. 2:**
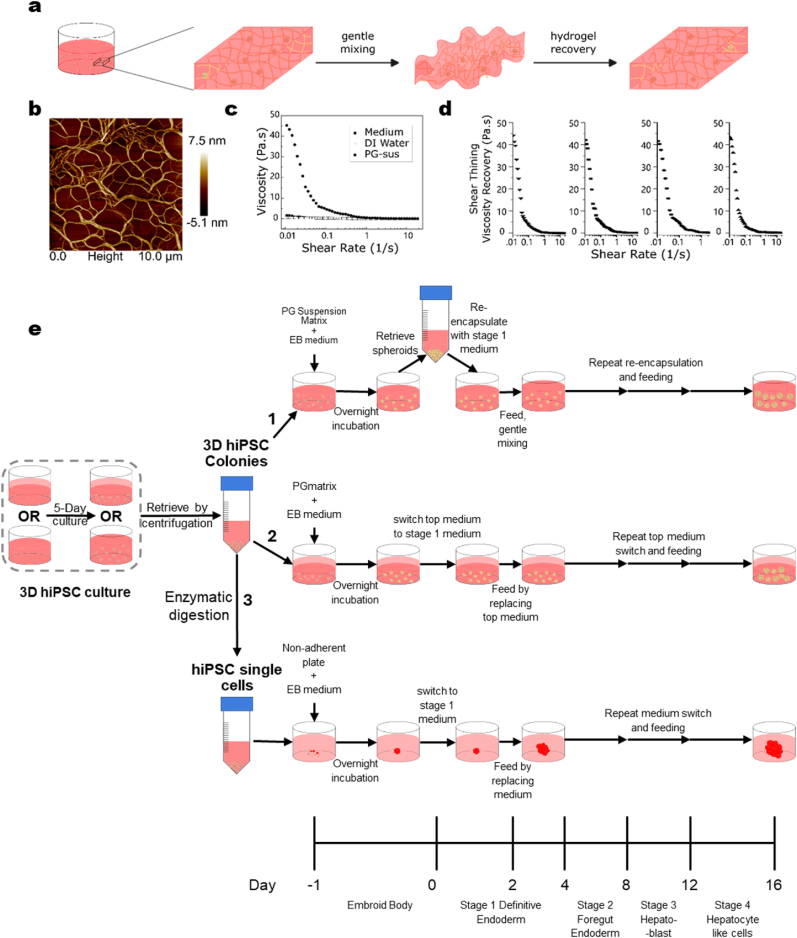
**Experimental workflow and design of a shear-thinning PepGel-suspension (PG-sus) hydrogel and development of a 3D differentiation protocol. a,** Schematic illustration of shear-thinning and self-healing (gel recovery) behaviors of PG-sus hydrogel during culture. **b,** AFM images of the PGmatrix suspension condition (PG-sus) nanoweb structure. **c,** PG-sus exhibited shear thinning behavior and turned into a liquid-like state after applying shear force. **d,** the shear-thinning PG-sus hydrogel can recover to a gel state after shear force removal, and the shear thinning–recovery process is repeatable. **e,** Schematic illustration of workflows, differentiation protocols and schedule of LC-hiPSC-derived hepatocytes; where Route 1 used LC-hiPSCs directly from a 5-day PG-sus subculture (gel strength <50Pa); Route 2 used LC-hiPSCs directly from a 5-day PG-embedded subculture (gel strength ∼500 Pa); and Route 3 used LC-hiPSCs from a 5-day PG-embedded subculture that were dissociated into single cells and re-aggregated into spheroids in a traditional suspension culture using non-adherent U-bottom 96-well plates.

## 2 Methods

### 2.1 3D culture of hiPSCs

***hiPSC 3D encapsulation and culture:*** hiPSCs derived from human foreskin fibroblasts (Applied StemCell, Inc., Milpitas, CA, USA) were maintained in 0.5% PGmatrix in PG-embedded culture media (PGmatrix 3D hiPSC Matrix Kit, PepGel, LLC, Winston Salem, NC USA). with mTeSR1 medium (StemCell Technologies, Cambridge, MA, USA) supplemented with the ROCKi (StemCell Technologies) following steps described by Li et al. [28]. In brief, hiPSCs, either as single cells or small clusters, were suspended in mTeSR1 medium. The cell suspension was then mixed with PGworks solution and PGmatrix 3D hiPSC Matrix solution from the PGmatrix 3D hiPSC Matrix Kit (PepGe LLC) According to the manufacturer's instructions, the cells were plated in a 24-well plate. After incubating at 37 °C for 30 min to allow for gelation, mTeSR1 medium (StemCell Technologies) supplemented with the 10 μM ROCKi was gently added to the top of the hydrogel. The medium was changed daily beginning on day 3, after which the cells were cultured for up to day 5. All PGmatrix-based 3D culture experiments in this study were conducted under stationary conditions without the need for shaking or agitation for culture volume of <300 mL per well or flask.

A U-shaped ultralow attachment 96-well (U96) plate (Corning, Glendale, AZ, USA) was used as a scaffold-free suspension method to generate hiPSC aggregates from the same culture medium mentioned above following the manufacturer's protocol.

***hiPSC 3D colony (spheroid) retrieval:*** Cells were harvested based on growth performance, typically on day 5 of each passage. At the end of each passage, the hydrogel was disrupted by thorough pipetting. The resulting mixture was then collected and transferred to a 50 mL centrifuge tube. The wells were rinsed with Dulbecco's phosphate-buffered saline (DPBS) without Ca^2+^/Mg^2+^ (Sigma‒Aldrich, Inc., St. Louis, MO, USA), and the solution was collected in a 50-mL tube. Additional DPBS was added to dilute the mixture more than 20-fold, followed by centrifugation at 200×*g* for 5 min.

***hiPSCs 3D culture in Matrigel:*** Single hiPSCs at 98% viability from subculture (i.e., 3D PGmatrix) were embedded in Matrigel (Corning) following the manufacturing using guide. Briefly, hiPSCs were resuspended in complete mTeSR Plus medium and mixed with ice-cold Matrigel at seeding density of 2 × 10^5^ cells/mL. The mixture was gently pipetted, plated at 125 μL per well in 48-well plates, polymerized at 37 °C for 1 h, and overlaid with 500 μL mTeSR Plus. hiPCS cells were then isolated on day 4 from Matrigel by using non-enzymatic Gentle Cell Dissociation Solution (STEMCELL Technologies, Cat. No. 100-0485) on ice for 40 min with gentle agitation (100 rpm), using a 30-fold excess volume relative to Matrigel. Then the hiPSCs spheroids were dissociated into single cells for further analysis or next passage. The same hiPSCs cells were embedded with PGmatrix following the methods in this section 2.1 as positive control.

***Quantification of hiPSC lumen structure:*** Method for lumen quantification was described in Supplementary Section 2.1.

### 2.2 Hepatic differentiation

***Differentiation preparation:*** In the 3D PepGel PGmatrix (PG-sus), after removing the supernatant, the hiPSC spheroids were directly resuspended in EB medium for hepatic differentiation within the hydrogel matrix. For U96 cell aggregate formation, hiPSC spheroids retrieved from 0.5% PG-embedded cultures were dissociated into single cells by resuspending them in 1x TrypLE (Thermo Fisher Scientific, Waltham, MA, USA) and incubating at 37 °C for 30 min. After incubation, mTeSR1 medium was added to stop the dissociation reaction, and the mixture was then centrifuged at 200×*g* for 5 min. Following removal of the supernatant, the hiPSCs were resuspended in EB medium for U96 cell plating and subsequent differentiation.

***Hepatocyte differentiation:*** The differentiation process followed the 4-stage protocol by Pettinato et al. [23] with some modifications. The overall process was illustrated in Fig. 1f. The day of plating was designated day 1. hiPSC spheroids or single cells were resuspended in EB medium (Supplementary Table 1), encapsulated in hydrogel or plated into U96 plates and then incubated at 37 °C overnight. On day 0, differentiation toward the definitive endoderm was initiated by the addition of activin A, basic fibroblast growth factor (bFGF) and transforming growth factor-beta (TGF-β) (PeproTech, Cranbury, NJ, USA). All differentiation supplements were purchased from PeproTech unless otherwise specified. By day 4, the medium was switched to EB medium supplemented with bone morphogenetic protein 4 (BMP-4) and fibroblast growth factor 4 (FGF4) to facilitate differentiation toward the foregut endoderm. Stage 3 was initiated on day 8 by switching to EB medium supplemented with Wnt inhibitory factor 1 (WIF-1) (R&D Systems, Minneapolis, MN, USA) and Dickkopf-related protein 1 (DKK-1) to induce hepatoblast differentiation. The final stage of hepatocyte maturation began on day 12 after supplementation with oncostatin M (OSM) and hepatic growth factor (HGF) and continued until day 16 (Fig. 1f). The medium was changed every other day throughout each stage.

The plating method varied among the different treatment groups. For differentiation into PG-sus cells, hiPSC spheroids were resuspended in EB medium and then mixed with PG-sus and PGworks at a seeding density of 1.5–1.6x10^4^ spheroids/mL (7500–8000 spheroids/well for 24-well plates). The volume ratio of the spheroid suspension to PG-sus to PGworks was 5.78:1:0.07 on day 1. The plate was then incubated at 37 °C.

For medium switching at the onset of each stage (days 0, 4, 8, and 12), spheroids were retrieved by diluting the hydrogel 2–3-fold with DPBS (no Ca^2+^/Mg^2+^) (Sigma‒Aldrich) and centrifuging at 700×*g* for 5 min. The retrieved spheroids were then resuspended in fresh medium for the next differentiation stage and mixed with PG-susceptible or PGworks at a ratio of 3.05:1:0.04. Every other day, fresh medium was added, and the mixture was gently mixed to ensure even dispersion (Fig. 1f).

For differentiation in U96 plates (aggregates), single hiPSCs were resuspended in EB medium and plated into U96 plates at 3.5x10^4^ cells/well. The morphologies of the cells at all differentiation stages were monitored with an Axio Vert A1 microscope (Carl Zeiss Microscopy, Germany).

***Hepatocyte differentiation efficiency-Flow cytometry:*** Single cells dissociated from hepatocyte-differentiated hiPSCs cultured in PG-sus, U96 aggregates, and LC-hiPSCs (control) cultured in PG-sus were fixed, permeabilized, and stained for Albumin and the nuclear transcription factor HNF-4α using a Human Stem Cell Transcription Factor Analysis Kit (BD Biosciences). Stained samples, along with unstained and isotype controls, were analyzed on a BD LSRFortessa X-20 flow cytometer. Data acquisition and analysis were performed using BD FACSDiva software v8.0.1 (BD Biosciences). Approximately 1 × 10^5^ cells were analyzed per sample. Experiments were performed in three technical replications.

### 2.3 Viscosity analysis

Viscosity measurements were conducted using a Kinexus lab + rheometer (Netzsch Instruments North America LLC, Burlington, MA, USA). A parallel plate configuration with a 20 mm plate diameter and 500 μm gaps was employed. The viscosity was assessed within a shear rate range of 0.01–20/s. The formed PG-sus hydrogel, deionized water, and pure medium were transferred to the lower measuring plate and allowed to stabilize for 20 min before viscosity was measured. The viscosity recovery of PG-sus was evaluated by repeating the viscosity measurement four times in total, with the hydrogel stabilized for 5 min between each measurement. The testing temperature was maintained at 37 °C. To prevent sample dehydration during the test, a thin layer of silicone oil was applied around the circumference of the sample.

### 2.4 Gene marker analysis by RT‒qPCR

Hepatocyte markers and functional gene expression were assessed through RT‒qPCR. Upon completion of the differentiation process, the 3D colonies were retrieved, and total RNA was extracted from the samples with a Direct-zol RNA MiniPrep Kit (Zymo Research Corp., Irvine, CA, USA) and diluted to 20 ng/μL. RT‒qPCR was conducted using the Bio-Rad CFX96TM Touch™ Real-time PCR Detection System and an iTaq Universal SYBR Green One-Step Kit (Bio-Rad, Hercules, CA, USA). The initial reverse transcription reaction was set at 50 °C for 10 min, followed by polymerase activation and cDNA denaturation at 95 °C for 1 min. Subsequently, the samples were subjected to 45 cycles of denaturation at 95 °C for 10 s, annealing at 58 °C for 30 s, and extension at 72 °C for 20 s. PSMB6 was used as a housekeeping gene [44]. The primer sequences for the target and housekeeping genes are available in Supplementary Table 2.

### 2.5 Immunofluorescence

PG-hiHs were retrieved and fixed using 10% neutral buffered formalin (NBF) (Sigma). Following fixation, the 3D colonies were rinsed with wash buffer (DPBS with Ca^2+^/Mg^2+^ supplemented with 0.2% Triton-X 100 and 0.1% gelatin from cold water (all from Sigma‒Aldrich) and subsequently incubated in 10% normal goat serum (Thermo Fisher) in wash buffer overnight for blocking. Next, the samples were incubated with primary antibodies (Supplementary Table 3) in blocking solution at 4°C overnight. Following three washes with wash buffer, the samples were incubated with the corresponding secondary antibodies (Supplementary Table 3) at 4 °C overnight, followed by nuclear staining using 4’,6-diamidino-2-phenylindole (DAPI) solution (Abcam, Cambridge, UK) for 1 h. Subsequently, the samples were subjected to three additional washes with wash buffer to remove excess reagent and then mounted onto glass slides for fluorescence imaging using a Zeiss LSM 700 confocal microscope (Carl Zeiss Microscopy).

For staining of polarity markers and bioprinted samples, the samples were initially fixed with 10% NBF, embedded in Tissue-Tek® O.C.T. Compound (Sakura Finetek USA, Inc., Torrance, CA, USA), and frozen at −20 °C. Using a Leica CM3050S cryostat (Leica Biosystems, Inc., Buffalo Grove, IL, USA), the samples were cryosectioned into slices measuring 50 μm (for bioprinted samples) and 14 μm (for polarity staining), which were then mounted onto glass slides. The slides were air-dried at 37 °C for 2 h prior to initiating the immunostaining process.

### 2.6 Histological processing and immunohistochemistry

Kansas State Veterinary and Diagnostic Laboratories (KSVDL) performed histological processing and immunohistochemistry. Initially, the cell spheroids were fixed overnight in 10% NBF, washed with DPBS supplemented with Ca^2+^/Mg^2+^ (Sigma), and subsequently embedded in 1% agar (Sigma‒Aldrich) solution cooled to approximately 60 °C. The agar-embedded samples were then sectioned and embedded in paraffin. These embedded samples were sliced, mounted onto glass slides, dewaxed, and subjected to staining with H&E.

For immunohistochemistry, 4-μm-thick sections were placed on positively charged slides and dried overnight. Antibody information is provided in Supplementary Table 4. The sections were dewaxed, rehydrated and subsequently heated using either a citrate-based (pH 6) or ethylenediaminetetraacetic acid (EDTA)-based (pH 9) epitope retrieval solution for 20 min. Hep Par 1 (Dako, Carpinteria, CA, USA), E-cadherin (Cell Signaling Technology, Danvers, MA, USA), claudin-5 (4C3C2), mouse monoclonal antibodies (Invitrogen, Waltham, MA, USA), and CK19 (Thermo Fisher) were incubated with the appropriate diluted antibodies for 15 min. After washing with BOND wash solution (Leica Biosystems, Inc.), the sections were incubated with alkaline phosphatase (AP)-labeled polymer anti-mouse antibody complex for 25 min and washed again with BOND wash solution. A refined red chromogen solution was applied, and the colored product was allowed to develop before counterstaining and mounting. The Ki-67 protocol utilizes a hydrogen peroxide block for 5 min, followed by incubation with the appropriate antibody for 15 min. After washing with BOND wash solution, the sections were incubated with polymeric horseradish peroxidase (HRP)-conjugated antibodies for 25 min and washed again with BOND wash solution. A diaminobenzidine chromogen solution was applied, and the colored product was allowed to develop before counterstaining and mounting. Normal canine liver tissue served as a positive control for Hep-Par1 cells. The normal canine small intestine served as a positive control for E-cadherin. A canine mast cell tumor served as a positive control for Ki-67. Normal canine skin served as a positive control for CK19. Immunostaining was performed using a BOND RX Research Stainer (Leica Biosystems, Inc.).

### 2.7 Oil Red O staining

Lipid storage was assessed using an Oil Red O staining kit (Abcam) according to the manufacturer's protocol, with modifications. hiPSC spheroids and PG-hiHs were fixed with 10% NBF for 1 h and then rinsed with DPBS containing Ca^2+^/Mg^2+^. Following fixation, the samples were resuspended in propylene glycol, plated into clear-well plates, and incubated at room temperature (RT) for 2 min, followed by a 6-min incubation with Oil Red O solution. The samples were then incubated with 85% propylene glycol for 1 min and washed twice with deionized water. Finally, stained samples were examined using an Axio Vert A1 microscope.

### 2.8 Periodic acid-Schiff (PAS) staining

Glycogen storage was evaluated using a periodic acid-Schiff (PAS) staining system (Sigma) according to the manufacturer's protocol, with modifications. HiPSC spheroids and PG-hiHs were fixed with 10% NBF for 1 h and subsequently rinsed with DPBS containing Ca^2+^/Mg^2+^. The fixed samples were then suspended in periodic acid solution and plated into clear-well plates for 5 min of oxidization at RT, followed by three washes with deionized water. Subsequently, Schiff reagent was applied, and the samples were incubated for 15 min at RT. After incubation, deionized water was added for 5 min to facilitate color development. Finally, the samples were examined using an Axio Vert A1 microscope.

### 2.9 Indocyanine green (ICG) uptake assay

This assay provided insight into the general metabolic activities of cells before and after hepatic differentiation. ICG powder (Sigma) was dissolved in DMSO (Sigma) at a concentration of 5 mg/mL. The resulting stock solution was aliquoted and stored at −80 °C. Prior to use, the stock solution was thawed and diluted with EB medium to achieve a final concentration of 1 mg/mL. The samples were retrieved from the hydrogel, washed with DPBS, and then resuspended in 1 mg/mL ICG solution, followed by incubation at 37 °C for 1 h. Subsequently, the samples were rinsed with DPBS before being imaged with an Axio Vert A1 microscope.

### 2.10 Intracellular urea content assay

The intracellular urea concentration was quantified using a colorimetric kit (Abcam) following the manufacturer's protocol. Briefly, samples containing approximately 2 × 10^6^ cells were first washed with cold PBS and then resuspended in assay buffer. Homogenization was achieved by passing the samples through a 31-gauge needle (Air-Tite Products Co., Inc., Virginia Beach, VA, USA). Following homogenization, the samples were centrifuged for 5 min at 3000×*g* to remove insoluble material, and the supernatant was collected and kept on ice. Standards, background, and test samples were prepared following the manufacturer's instructions in a 96-well plate and subsequently incubated at 37 °C in the dark for 1 h. Absorbance readings were obtained at 570 nm using a SpectraMax Plus Absorbance Microplate Reader (Molecular Devices, LLC., San Jose, CA, USA). Standard curve was established using standards recommended by manufacturer's protocol. Sample readings, after subtraction of backgrounds, were converted to urea contents using the standard curve.

### 2.11 Cytochrome P450 activity assessment

The activity of cytochrome P450 (CYP) enzymes was assessed to evaluate hepatocyte detoxification functions. Promega P45-Glo kits (Promega, Madison, WI, USA) were used to evaluate the functions of CYP3A4 and CYP1A2 following the manufacturer's instructions. Upon completion of hepatic differentiation, hiH colonies were retrieved and plated in triplicate into a U96 plate with approximately equal cell numbers. These samples were then incubated with EB medium supplemented with 0.1% DMSO (as a control), 50 μM omeprazole (for CYP1A2), or 20 μM rifampicin (for CYP 3A4) at 37 °C for 72 h, after which the medium was changed every 24 h. Following drug induction, the samples were washed 3 times with DPBS and then incubated with the corresponding CYP substrates for 1 h. Subsequently, 25 μl/well of the incubated solution was extracted and mixed with Luciferin Detection Reagent. Moreover, the remaining samples were mixed with CellTiter-Glo Cell Viability Assay reagent (Promega). The plate was then placed on a Fisherbrand microplate shaker (Fisher Scientific) for 5 min of mixing at 1000 rpm, followed by incubation in the dark at room temperature for 20 min before luminescence was measured using a GloMax Navigator luminometer (Promega). The enzyme activity level was calculated as follows: Enzyme activity level = P45-Glo reading/CellTiter-Glo reading × 10^6^. The calculation results represent enzyme activity per live cell.

### 2.12 Image staining intensity quantification

Image quantification was performed using Fiji-ImageJ (version 68) [45]. Briefly, fluorescence or stained images were split into individual color channels, background subtracted, and thresholds adjusted. Fluorescence or dye intensities were quantified as the mean gray value for each channel. For intensity distribution analysis, a line parallel to the bottom edge of the image was drawn through the center of each spheroid, and the intensity profile along this line was recorded.

For Ki67^+^ cell analysis, cell counts rather than mean gray values were used. For Oil Red O, PAS, and ICG staining, the mean gray values were measured from selected target spheroids instead of whole-image analysis. All measurements were performed in triplicate. Mean gray values were used to generate intensity graphs, and relative intensity was calculated as:

$$\text{Relative}\;\text{intensity}=\frac{Mean\;gray\;value\;of\;target\;channel}{Mean\;gray\;value\;of\;nuclei\;\left(DAPI\right)\;channel}$$

$$\text{Live}/\text{Dead}\;\text{ratio}=\frac{Mean\;gray\;value\;of\;live\;cells\;\left(green\right)}{Mean\;gray\;value\;of\;dead\;cells\;\left(red\right)}$$

### 2.13 Live shipping of hiPSCs and PG-hiH spheroids

The live hiPSC spheroids and PG-hiHs isolated from 3D PGmatrix cultures (either PG-embedded or PG-sus) were encapsulated within 0.2% PGmatrix-LiveShip hydrogel (PepGel LLC) following the same procedure for hiPSC culture encapsulation. The encapsulation density was 20,000–25,000 spheroids/mL for hiPSCs and approximately 18,000–20,000 PG-hiH 3D colonies/mL (approximately 3 million cells/mL). One milliliter of the encapsulation mixture was then transferred to a 5 mL conical vial and incubated at 37 °C for 30 min to allow for hydrogel formation. Approximately 4 mL of complete mTeSR1 media supplemented with the ROCK inhibitor Y27632 (Stemcell Technologies) or hepatocyte maintenance medium (Thermo Fisher) was then added to the top of the gel to fill the vial for two purposes: feeding the cells and expelling air from the vial to minimize the formation of air bubbles during transportation. The conical vials were carefully sealed with parafilm (Fisher Scientific), thoroughly wrapped, and positioned in a thermal box overnight shipping at ambient temperature. Upon receipt, spheroids were retrieved from 3D culture using previously described methods for hiPSC retrieval.

### 2.14 In vivo animal study

***Animal study******:*** All animal studies and procedures were conducted in accordance with the ethical guidelines established by the National Institutes of Health animal care and use and were approved by the University's Animal Care and Use Committee (201709890). The experimental and control animals were housed under identical pathogen-free conditions. Briefly, 8- to 12-week-old immunocompromised male NSG (NOD. Cg-Prkdc^scid^Il2rg^tm1Wjl^/SzJ) mice, purchased from Jackson Laboratory (Bar Harbor, ME, USA), were divided into 4 cohorts with 5 mice per group. Adequately anesthetized mice underwent an incision to access the peritoneum, exposing the spleen and splenic vein. A suspension of 100 μl of PG-hiHs harboring approximately 1x10^6^ cells, 1x10^6^ PHHs or PepGel hybrid PGmatrix-CDX hydrogel (PepGel LLC) was injected with a 27G needle and delivered to the liver via the splenic vein. Seventy-two hours after injection, the mice were subjected to an established model of warm hepatic ischemia‒reperfusion injury. In this procedure, the arterial and portal venous vessels supplying blood to the cephalad lobes (∼70%) of the liver were interrupted with an atraumatic clip for 30 min. Following this ischemic period, the clip was removed, and the lobes were reperfused. A 50-μL blood sample was retrieved from the tail vein at 72 h and 5 days after the injection for further analysis. Throughout all procedures, the mice received appropriate multimodal analgesic regimens, including meloxicam and buprenorphine, to ensure their welfare. On day 8 after injection, the mice were euthanized, and blood and liver tissue were collected for subsequent analysis.

***Human serum ALB ELISA:*** Human serum ALB was quantified using the DuoSet ELISA Kit (R&D Systems) strictly according to the manufacturer's instructions. Serum samples obtained by centrifugation of heparinized blood were diluted 1:100 in sample diluent. The absorbance of each well was measured immediately after the addition of STOP solution using a SpectraMax M3 microplate reader (Molecular Devices, San Jose, CA, USA) set to 450 nm. The data are presented in ng/mL of serum.

***Human serum alpha-1 antitrypsin:*** Quantification of human alpha-1 antitrypsin (A1AT) in mouse serum samples was achieved with an indirect sandwich ELISA method. Briefly, the wells were coated overnight at 4 °C with Anti-human aplha-1-antitrypsin goat antiserum (MP Biomedicals, Solon, OH, USA) diluted 1:200 in Voeller's buffer. Following the washing steps, the samples and standards were added at 100 μl/well and incubated for 2 h at 37 °C. Subsequently, the wells were washed again and incubated with 100 μl of rabbit AAT-1 antibody (Dako, Santa Clara, CA, USA) diluted in 0.2% bovine serum albumin (BSA)/washing buffer. This was followed by incubation with horseradish peroxidase-conjugated goat anti-rabbit IgG (Bio-Rad. Hercules, CA, USA). Finally, 100 μl of Quanta blue fluorogenic peroxidase substrate (Thermo Fisher) was added to each well to initiate the ELISA. Fluorescence readings were taken at 325/420 nm using a SpectraMax M3 microplate reader (Molecular Devices). The data is presented as pM (picomolar) of human A1ATl in mouse serum.

***Immunohistochemistry:*** Liver tissues were fixed in formalin and embedded in paraffin (FFPE). Five-micron-thick sections were cut using a minux microtome (RWD Life Science, Sugar Land, TX, USA) and affixed to slides. These sections were then deparaffinized, rehydrated, and washed before heat-induced antigen retrieval with 10 mM citrate buffer (pH 6). Subsequently, the sections were blocked using a solution of 3% water and 2.5% horse serum, followed by overnight incubation at 4 °C with primary antibodies (Supplementary Table 4). After washing, the sections were incubated with ImmPRESS horse anti-rabbit IgG (Vector Laboratories, Inc., Newark, CA, USA), followed by chromogen development using 3,3′-diaminobenzidine (DAB) (Vector Labs) and counterstaining with hematoxylin (Sigma). Finally, the slides were analyzed using a Nikon Eclipse 90i Microscope (Nikon).

### 2.15 Bioink formulation preparation

Two hundred milligrams of polyethylene glycol diacrylate (PEGDA) powder (6000 MW) (Advanced Biomatrix, Carlsbad, CA, USA) was dissolved in 1 mL of deionized water to prepare a 10% stock solution. A 3% stock lithium phenyl-2,4,6-trimethylbenzoylphosphonate (LAP) (Sigma-Aldrich) solution was prepared by dissolving 20 mg of powder in 0.667 mL of deionized water. Both stock solutions were sterilized by filtering through a 0.2 μm filter. To prepare the bioink solution, the PEGDA solution was added to the PepGel hybrid (PGS) (PGmatrix Spheroids Kit, PepGel, LLC) solution and thoroughly mixed, followed by the addition of cell medium. Finally, LAP was added to the solution to achieve a final concentration of 0.2%. The bioink was shielded from light to prevent premature crosslinking before UV curing. The different ratios of PEGDA-PGS solutions used are detailed in Supplementary Table 5.

### 2.16 3D bioprinting of hiPSC spheroids and hiH 3D colonies

***DLP bioprinting:*** A bioink containing 5% PEGDA and 2% PGS (Supplementary Table 5) with the photo initiator LAP (0.2%) was placed into the vat before bioprinting, after which the platform was reset to its initial state. Subsequently, the bioink was crosslinked on the platform in a layer-by-layer manner by adjusting the pattern displayed from the projector with a light density of 18.6 mW/cm^2^. The platform was then gradually raised after each completed layer until the entire construct was produced. Three-dimensional structures, such as the ear, nose, and brain, were printed at 50 μm thickness per layer after 20 s of exposure for each layer. For 2D patterns such as those of the heart, lung, and lobule, the bioink was transferred onto an oxygen plasma-cleaned glass slide and exposed for 25 s. Finally, the 3D structures were visualized using a painting pigment (Easyou, China), while rhodamine B (Sigma) was used for visualizing the 2D patterns.

***Cell viability (live/dead staining):*** Live/dead staining (Thermo Fisher) was used to assess the viability of cells in the printed constructs. Following rinsing with PBS, the bioprinted constructs were submerged in a solution containing 2-μM calcein-AM and 4-μM ethidium homodimer-1 and then incubated for 35 min. Subsequently, the constructs were observed via fluorescence microscopy to visualize the stained cells.

### 2.17 Statistics

Statistical analysis was performed with JASP ver. 0.18.1 (The JASP Team). Group comparisons were performed using ANOVA, followed by post hoc tests with Tukey correction, with a 95% confidence interval.

## 3 Results

### 3.1 Synthetic peptide hydrogels enabled hiPSCs to self-organize into LC in 3D colonies

We discovered that hiPSCs formed distinct 3D LC-hiPSC structures within their colonies in synthetic peptide hydrogel (Fig. 1a and b), reflecting intrinsic pluripotency, whereas the aggregates, formed under scaffold-free suspension condition, exhibited solid spheroid morphology (Fig. 1a–d). The rosette-like cytoskeletal LC structure was clearly demonstrated by F-actin and α-tubulin-stained images and patterned fluorescence signal intensity distributions, indicating that the 3D LC-hiPSC colonies possessed highly self-organized LC structures (Fig. 1b and c, Supplementary Fig. 1a). In contrast, this self-organized structure was absent in the hiPSC aggregates (Fig. 1d and e, Supplementary Fig. 1b). This finding suggested that the LC-hiPSCs potentially possessed physiologically relevant differentiation capabilities for generating more complex structures toward somatic cells [1,2,27,46,47].

Most importantly, more than 95% of cells formed LC-hiPSC (Supplementary Fig. 1d and e), and proliferated robustly and were maintained long term in this 3D system, achieving a 10-20-fold expansion, depending on cell type and dissociation enzyme, with an average viability of ∼97% across multiple passages, as reported in our previous work [28], while lumen architecture is explicitly characterized in the present study. Furthermore, the formation of the LC structure was significantly influenced by 3D culture conditions. hiPSCs developed symmetrical and spherical LC structures in PepGel hybrid (PGmatrix) 3D cultures when using either mTeSR1 or mTeSR plus complete medium (Supplementary Fig. 2a). In contrast, when cultured in essential 8 (E8) medium, hiPSC also formed LC structures; however, some cavities were less symmetrically spherical (Supplementary Fig. 2b), likely due to the reduced presence of growth factors compared to the growth factor-rich mTeSR medium. When no growth factors were present in the medium, such as in DMEM, hiPSCs ceased proliferation entirely (Supplementary Fig. 2c).

Notably, matrix composition also played a critical role in structural integrity. Incorporating even a small amount of vitronectin protein into PGmatrix, despite the presence of mTeSR plus medium, led to structural alterations, with cavity formation nearly disappearing at 1% vitronectin protein content (Supplementary Fig. 3). Although Matrigel is widely regarded as a natural ECM matrix, it is derived from mouse tumors. Under Matrigel culture conditions, LC-hiPSCs rapidly collapsed by day 3 in passage one and were completely lost by passage 2, whereas more than 95% of cells maintained and formed lumen structures in PGmatrix (Supplementary Fig. 4). In short, the PGmatrix 3D culture condition presented here was uniquely identified for LC-hiPSCs maintenance and expansion.

### 3.2 3D LC-hiPSCs exhibited exceptional differentiation capability as demonstrated by hepatocytes differentiation in a scalable synthetic peptide hydrogel

In this study, Matrigel and vitronectin were initially evaluated for 3D LC-hiPSC culture, as both are widely used ECM substrates for hiPSC maintenance in 2D. However, neither Matrigel nor vitronectin supported stable 3D lumen formation in hiPSCs (Supplementary Figs. 3 and 4). In contrast, PGmatrix robustly supported LC-hiPSC formation (Fig. 1). Therefore, PGmatrix was selected as the sole hydrogel platform for this study to enable a clear and direct comparison with conventional hiPSC spheroid aggregates, as suspension-based spheroid aggregation remains the most commonly used workflow for hiPSC-derived organoid differentiation.

To enable scalable 3D scaffolding for LC-PSC generation and hepatocyte differentiation, based on our pilot studies on hepatocyte differentiation (Supplementary Fig. 5), the modified PGmatrix [28] was developed into a PG 3D suspension matrix system (PG-sus) (Fig. 2). This PG-sus system exhibits self-assembly and mechanical properties comparable to those of PGmatrix embedded conditions reported previously by us, forming a nanofiber network (Fig. 2b) that supports LC-hiPSCs formation as in a 3D embedded environment, while remaining fluid-like during fresh medium addition and mixing. Once mixing stops, the matrix spontaneously recovers its 3D embedded state with a static viscosity of about 55 Pa s due to its shear-thinning and self-healing properties (Fig. 2a and 2c-d). Such sol–gel transition of this hydrogel remained repeatedly reversible within the limits of our testing. However, whether this reversibility will diminish over extended cycles remains unclear. For medium exchange or cell harvesting, PG-sus behaves like water under centrifugal shear, allowing cells to be pelleted at the bottom (Fig. 2c). The integrity of 3D LC-hiPSCs cultured in PG-sus was validated, demonstrating stable proliferation, high viability, and sustained lumen formation comparable to embedded conditions across multiple passages (Fig. 1d and e; Supplementary Fig. 1b and e).

The experimental workflows and the 4-stage hepatocyte differentiation protocol for three treatment routes are illustrated in Fig. 2e. Route 1 used LC-hiPSCs directly from a 5-day PG-sus subculture (gel strength <50Pa); Route 2 used LC-hiPSCs from a 5-day PG-embedded subculture (gel strength ∼500 Pa); and Route 3 used LC-hiPSCs directly from a 5-day PG-embedded subculture that were dissociated into single cells and re-aggregated into spheroids in a traditional suspension culture using non-adherent U-bottom 96-well plates.

Stage 1 differentiation began with encapsulation of LC-hiPSCs in PG-sus for EB induction, followed by switching to Stage 1 differentiation medium (Fig. 2e, Route 1). In this process, LC-hiPSCs were retrieved directly from the 5-day 3D PG-sus culture. Medium switching from EB to Stage 1 was performed by centrifugation, after which the EB pellet was re-encapsulated in PG-sus with Stage 1 medium. Cell feeding was carried out by blending fresh Stage 1 medium with the hydrogel culture. Stages 2-4 followed the same workflow, repeating the centrifugation-based medium switching. This design allows the PG-sus system to be readily scaled up in static culture flasks up to the liter scale, with potential for further expansion using stirred-tank bioreactors, bioprinting, or microfluidic systems.

For Routes 2 and 3, the same differentiation workflow was applied, except that medium switching and feeding were performed by replacing the top medium (Route 2) or the culture medium (Route 3), respectively (Fig. 2e, Routes 2 and 3).

**Fig. 3:**
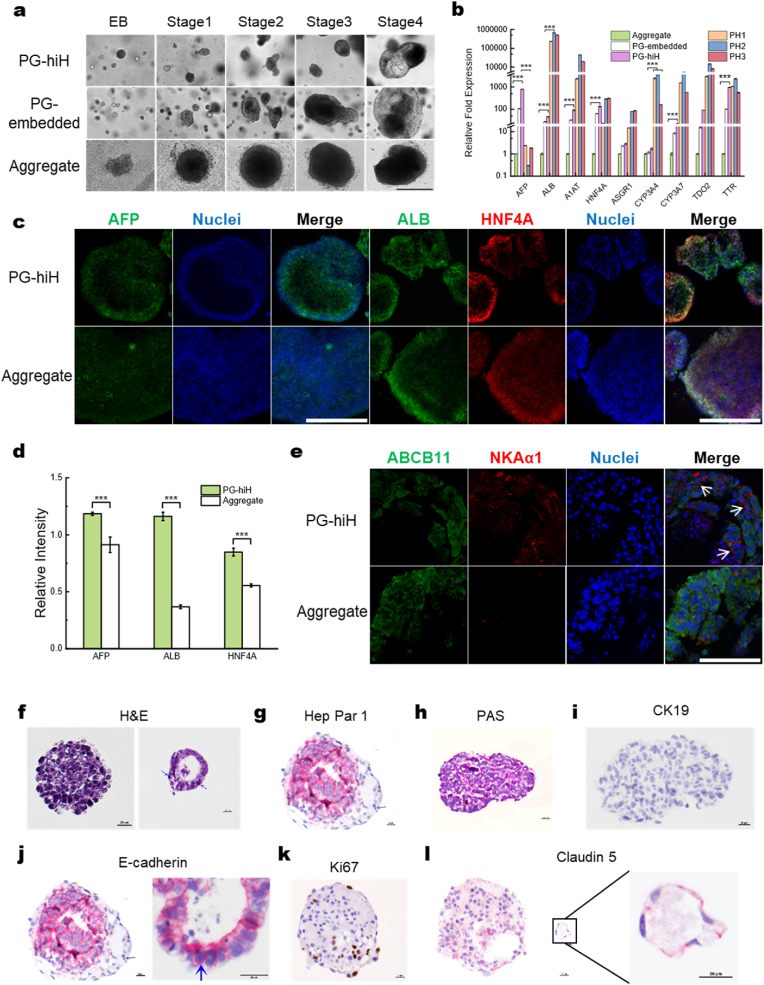
**LC-hiPSC 3D colonies were differentiated into PG-hiH 3D colonies, which exhibited hepatocyte characteristics. a**, Changes in the morphology of 3D colonies differentiating in different microenvironments: encapsulated in PepGel PGmatrix 3D suspension (PG-sus) following Route 1; embedded in PepGel PGmatrix hiPSC hydrogel (PG-embedded) following Route 2; and 3D colonies formed from single cells in a nonadherent suspension (Aggregate) following Route 3. Scale bar, 600 μm **b,** RT‒qPCR results showing the fold change in expression of hepatocyte marker genes in PG-hiH, PG-embedded and Aggregate in comparison to those in three PHH samples. The Aggregate data were used as a control, and the results are shown as the mean ± SEM (standard error of the mean) (n = 3); ∗p < 0.05; ∗∗p < 0.01; ∗∗∗p < 0.001. **c,** The PG-hiH, PG-embedded and Aggregate groups expressed the hepatocyte marker proteins AFP, ALB and HNF4A. Scale bar, 400 μm **d,** Quantification of AFP, ALB and HNF4A intensity from Fig. 3c, relative intensity = Target/DAPI, each image was measured three times, data shown as mean ± SEM (n = 3), ∗p < 0.05; ∗∗p < 0.01; ∗∗∗p < 0.001. **e,** PG-hiH and Aggregates were sectioned and stained for the polarity markers ABCB11 and NKAα1. Arrows indicate cells with both markers on different sides. Scale bar, 100 μm. **f,** Representative morphologies of PG-hiH colonies after sectioning and staining with H&E, showing hepatic plates and mitotic cells (arrows). Scale bar, 20 μm **g,** PG-hiHs showed strong Hep Par 1 expression (red). Scale bar, 20 μm **h,** PG-hiHs were stained with PAS to highlight intracytoplasmic glycogen stored in hepatocytes and hepatoblasts (purple). Scale bar, 20 μm. **i,** PG-hiHs were negative for cytokeratin 19 (CK19), a marker protein for cholangiocytes, indicating complete differentiation toward hepatocytes. Scale bar, 20 μm. **j,** Staining for E-cadherin showed the presence of tight junctions between hepatocytes in PG-hiHs (arrows). Scale bar, 20 μm. **k,** Several cells in PG-hiHs were positive for Ki67 (brown), indicating proliferation ability. Scale bar, 20 μm **l,** Claudin 5 staining of endothelial cells (red) with elongated nuclei surrounding the central lumen resembling a primitive hepatic sinusoid. Scale bar, 20 μm.

LC-hiPSCs derived hepatocytes exhibited superb gene marker expression and polarized luminal morphologic structures in either PG-sus or PG-embedded systems compared to aggregate-derived hepatocytes formed in scaffold-free suspension. The LC-hiPSCs uniformly expanded within PG-sus throughout the 4-stage hepatocyte differentiation process, evolving into physiologically grown human induced hepatocyte 3D colonies (PG-hiH) (Fig. 3a). PG-embedded hepatocytes also follow a growth pattern similar to that observed in PG-hiH. In contrast, the hiPSC spheroids from the scaffold-free suspension system began growing but remained as a single aggregate, ultimately differentiating into a large, dense hepatocyte aggregate lacking apparent structural heterogeneity (Fig. 3a).

RT‒qPCR analyses of 16-day-old PG-hiHs revealed that the PG-hiHs exhibited significantly greater expression of hepatocyte marker genes than did those in aggregates. Additionally, all the tested PG-hiH colonies outperformed those from the PG-embedded subgroup. Moreover, the PG-hiHs had expression levels of *HNF4A* (p = 0.615) and *TTR* (p = 0.813) comparable to those in PHHs (Fig. 3b). *HNF4A,* a crucial transcription factor enriched in the liver, is critical for hepatocyte differentiation and maintenance [48,49]. Its expression increases when hiPSC-derived definitive endoderm differentiates toward the hepatic fate [48,49], which coincides with our PCR analyses, which revealed an increase in *HNF4A* expression. *TTR,* a transporter for thyroxine and vitamin A primarily produced in the liver [50], also exhibited elevated expression. Moreover, compared with those in PHHs, the PG-hiH exhibited higher levels of *AFP* and relatively lower levels of *ALB* and *CYP3A4*, indicating a premature state.

Flow cytometric analysis of PG-hiH and U96 aggregates using albumin and HNF-4α as markers (Supplementary Fig. 6, Table 5) was performed to assess differentiation efficiency. PG-hiHs exhibited markedly higher marker expression than those in U96 aggregates: 95 % vs. 78% albumin-positive cells and 40% vs. 31% HNF4A-positive cells, respectively. Undifferentiated hiPSCs used as control did not express HNF4A, confirming the absence of hepatic differentiation, although ∼26.3% showed albumin-positivity, likely due to bovine serum albumin (BSA) present in mTeSR1 medium, which can be internalized or surface-bound, as previously reported. These results indicate that PG-sus culture enhances hepatic differentiation efficiency relative to U96 aggregates, consistent with prior reports demonstrating that physiologically relevant 3D microenvironments promote hepatocyte maturation through improved cell–cell and cell–matrix interactions [23,51].

Further, immunochemical staining of both PG-hiH and hepatocyte aggregates for the AFP, ALB, and HNF4A proteins in 16-day-old differentiated hepatocytes revealed the expression of these characteristic proteins in both types of hepatocytes (Fig. 3c). However, these proteins were evenly distributed within the PG-hiHs, clearly outlining the luminal structures (Fig. 3c) and Z-stack images (Supplementary Fig. 7 top row). In contrast, for the 16-day-old hepatocyte aggregates, the proteins were predominantly concentrated on the surfaces and no pattern in the spheroid center: the surface image (Supplementary Fig. 7 bottom left) was greener and redder, while the image toward the center of the aggregate (Supplementary Fig. 7 bottom right) was bluer, as observed from the Z-stack images (Supplementary Videos 1, 2).

Supplementary video related to this article can be found at https://doi.org/10.1016/j.bioactmat.2026.03.029

The following isare the supplementary data related to this article.

Multimedia component 3 Multimedia component 3

Multimedia component 4 Multimedia component 4

Moreover, the multi-luminal structures observed in PG-hiHs (Fig. 3c, Supplementary Fig. 7) can be attributed to hepatocyte polarity [52], which induces morphological integrity of tight junctions and apical plasma membranes, facilitating the formation of microvilli and bile canaliculi networks to support liver functions [52]. Confocal microscopy revealed that hepatocyte aggregates produced the apical membrane protein ATP-binding cassette subfamily B member 11 (ABCB11) on most cells but expressed the basolateral protein α1 Na+/K + -ATPase (NKAα1) at low levels (Fig. 3e). Conversely, PG-hiH expressed elevated levels of NKAα1 in addition to ABCB11, as shown by the merged image showing colocalization of the two proteins on different sides of some cells (Fig. 3e arrows), indicating the development of polarity in PG-hiHs, contributing to the formation of multi-luminal morphological structures.

Next, the PG-hiHs were embedded in 1% agar and subjected to immunohistochemical analysis. We found that the PG-hiH spheroids exhibited varying thicknesses accompanied by occasional mitotic activity and individual cell apoptosis. These 3D PG-hiH colonies were categorized into two types: solid hepatocyte spherical 3D colonies showcasing a primitive hepatic cord-like arrangement with primitive hepatic sinusoids and hollow spherical 3D colonies (Fig. 3f), and some PG-hiHs exhibited distinctive hexagonal lobular architecture. The largest cross section of the PG-hiH 3D colonies consisted of sixty cells, occupying a total area of 93.32 μm^2^. PG-hiH 3D colonies were quantitively observed to comprise mature hepatocytes expressing hepatocyte paraffin 1 (Hep Par 1) (Fig. 3g, Supplementary Fig. 8a) and accumulating intracytoplasmic glycogen (Fig. 3h, Supplementary Fig. 8a). No ductular cells were detected, as the colonies universally tested negative for the cholangiocyte marker cytokeratin 19 (CK19) (Fig. 3i). Tight junctions were evident in both the solid and hollow spheres as indicated by granular or segmental E-cadherin immunoreactivity along the basolateral membrane (Fig. 3j). Approximately 17% of the constituent hepatocytes were proliferating, as evidenced by nuclear immunoreactivity and the quantification of staining intensity for the nonhistone, nuclear protein Ki-67 (Fig. 3k, Supplementary Fig. 8b).

Overall, the PG-sus 3D system provided a conducive microenvironment supporting the formation of physiologically relevant LC-hiPSCs further transformed into polarized multi-luminal PG-hiH 3D colonies resembling primitive hepatic lobule-like structures. hiPSCs in PG-sus exhibited significantly higher differentiation efficiency (95% albumin, 40% HNF4A) compared to in U96 aggregate (78% albumin and 31% HNF4A) (Supplementary Fig. 6, Table 5).

### 3.3 LC-hiPSC-derived PG-hiHs showed intriguing functional properties in vitro

**Fig. 4:**
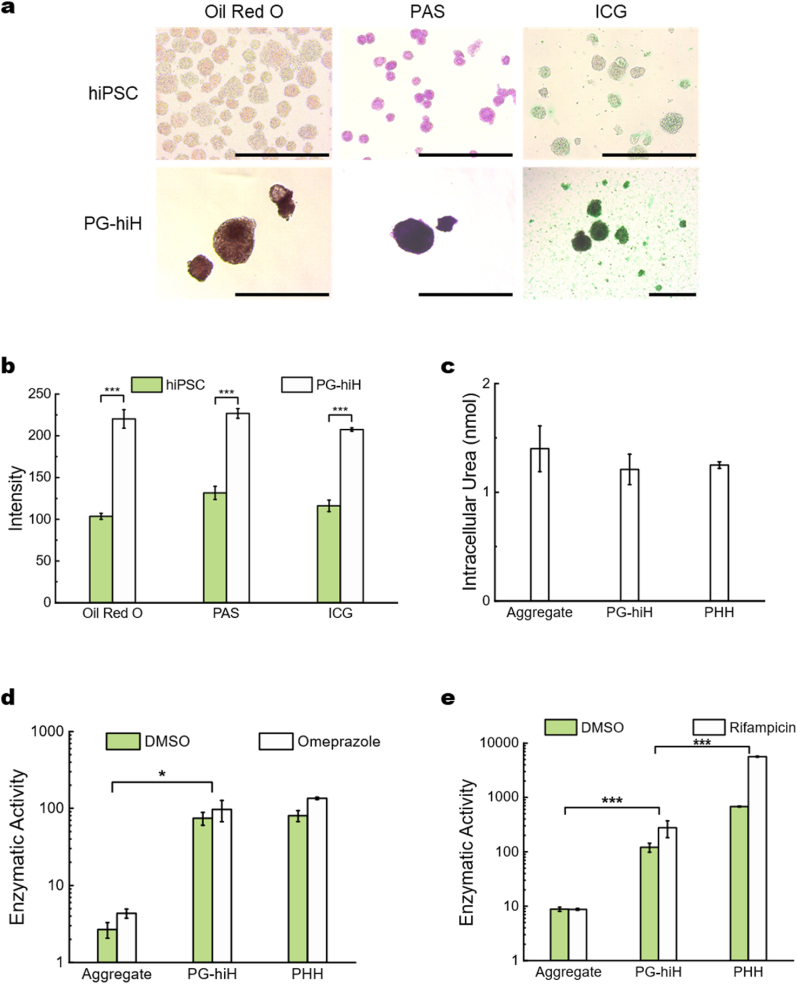
**PG-hiHs exhibited superior *in vitro* hepatocyte functions. a,** Compared to undifferentiated hiPSC spheroids, PG-hiHs stored neutral lipids and glycogen according to Oil Red O and PAS staining, respectively. In addition, PG-hiHs were capable of ICG uptake, demonstrating basic metabolic functions. Scale bar, 600 μm **b,** Quantification of dye intensity in Fig. 4a. Spheroids in the images were picked and measured separately, data shown as mean ± SEM (n ≥ 3 for all groups), ∗p < 0.05; ∗∗p < 0.01; ∗∗∗p < 0.001. **c,** Intracellular urea content measured by a colorimetric assay showed similar urea production by PG-hiHs and hiH Aggregate in comparison to that of PHHs. The results are shown as the mean ± SEM (n = 3). **d-e,** Activity levels of CYP enzymes in hiH Aggregate and PG-hiHs compared to those in PHHs induced with DMSO (control) or the specific inducers omeprazole for CYP1A2 (**d**) or rifampicin for CYP3A4 (**e**). Enzyme activity level = CYP assay luminescence intensity/viability luminescence intensity × 10^6^. The results are shown as the mean ± SEM (n = 3), ∗p < 0.05; ∗∗p < 0.01; ∗∗∗p < 0.001.

The nutrient storage ability, metabolic properties, and cytochrome P450 (CYP) activity of the PG-hiHs were characterized in vitro. Oil Red O staining intensity of PG-hiHs differentiated from PG-sus highlighted the proficiency of these cells in neutral lipid storage [53] (Fig. 4a and b). hiPSC spheroids served as the control prior to differentiation. The differentiated PG-hiHs exhibited dark purple high staining intensity with periodic acid–Schiff (PAS) staining, indicating the presence of abundant glycogen [54] (Fig. 4a and b). ICG uptake intensity by PG-hiH colonies confirmed the occurrence of hepatocyte-specific metabolic functions [55] (Fig. 4a and b). Ammonium metabolism is often evaluated through urea secretion into the medium following the incubation of hepatocytes with ammonium chloride [56]. However, this method requires precise cell viability measurements for accurate calculation. Despite the similar cell numbers and viability of the tested hepatocytes, changes in viability during the necessary 24-h incubation introduce variability to the final calculation. Therefore, we quantified the intracellular urea concentration as an indicator of ammonia metabolism and urea production using a colorimetric assay for both PG-hiH colonies and hiH aggregates as well as PHHs. The results indicated no significant differences in urea generation among hepatocytes from aggregate, PG-hiH, or PHHs (Fig. 4c), suggesting comparable levels of urea production.

To evaluate the detoxification capacity of the samples, we assessed the activities of two representative P450 enzymes found in the liver: CYP1A2 and CYP3A4 [23,57]. CYP1A2 represents a major component of the human CYP1A subfamily and is the last major CYP enzyme involved in maturation after birth [58]. Its activity is essential, serving as the primary clearance mechanism for approximately 10% of medications, such as clozapine, duloxetine, and propranolol, as well as caffeine and endogenous compound, such as melatonin [59]. Conversely, CYP3A4 is the most abundant and significant CYP enzyme involved in more than 40% of phase I metabolism [60]. Accounting for approximately 20% of liver CYPs, CYP3A4 plays a vital role in the metabolism of nearly 50% of drugs [61]. Consequently, measuring the activities of CYP1A2 and CYP3A4 can provide insight into a significant portion of hepatocyte CYP activities, offering information on the detoxification capacity of hepatocytes. The basal activity levels of these enzymes were assessed using dimethyl sulfoxide (DMSO) as the inducer. Notably, compared with hepatocyte aggregates, PG-hiHs exhibited significantly greater basal activity for both CYP1A2 (*p* = 0.009) and CYP3A4 (*p* = 0.005) (Fig. 4d and e). Upon induction, CYP1A2 activity increased across all three groups, but hepatocytes from the aggregates had significantly lower induction activity levels than did PG-hiHs p (*p* = 0.022). Interestingly, the CYP1A2 activity in PG-hiHs was similar to that in PHHs, regardless of sex (*p* = 0.926 and *p* = 0.333, respectively). While the PG-hiHs exhibited lower CYP3A4 activity than the PHHs did (*p* < 0.001), their basal activity levels were notably greater than those of the aggregates (*p* = 0.005). Conversely, compared with PHHs, hepatocyte aggregates displayed significantly lower CYP activity both with and without induction (*p* < 0.001). These findings suggest that PG-hiHs exhibit a markedly greater degree of maturation than hepatocyte aggregates.

### 3.4 Live-shipping of LC-hiPSCs and PG-hiHs

PG-hiH colonies obtained from the 3D PG-sus system were shipped alive under ambient conditions to collaborative institutions for in vivo and 3D bioprinting studies. This approach was chosen since cryopreservation significantly reduces hepatocyte properties [62,63] and causes variability in experimental results. To ensure shipping quality, the changes in quality of the LC-hiPSC 3D colonies were initially evaluated after live shipping (Supplementary Fig. 9). The results indicated that, compared with freshly harvested LC-hiPSCs, those underwent ambient shipping maintained more than 96% viability (Supplementary Fig. 9a), proliferated well in 2D culture (Supplementary Fig. 9b) and retained their pluripotency, as shown by tri-lineage differentiation assays (Supplementary Figs. 9c, d, e). Consequently, PG-hiH spheroids were shipped to collaborative institutions using the same shipping protocol.

### 3.5 In vivo functional properties of PG-hiHs were comparable to those of PHHs after transplantation into immunocompromised mouse livers

**Fig. 5:**
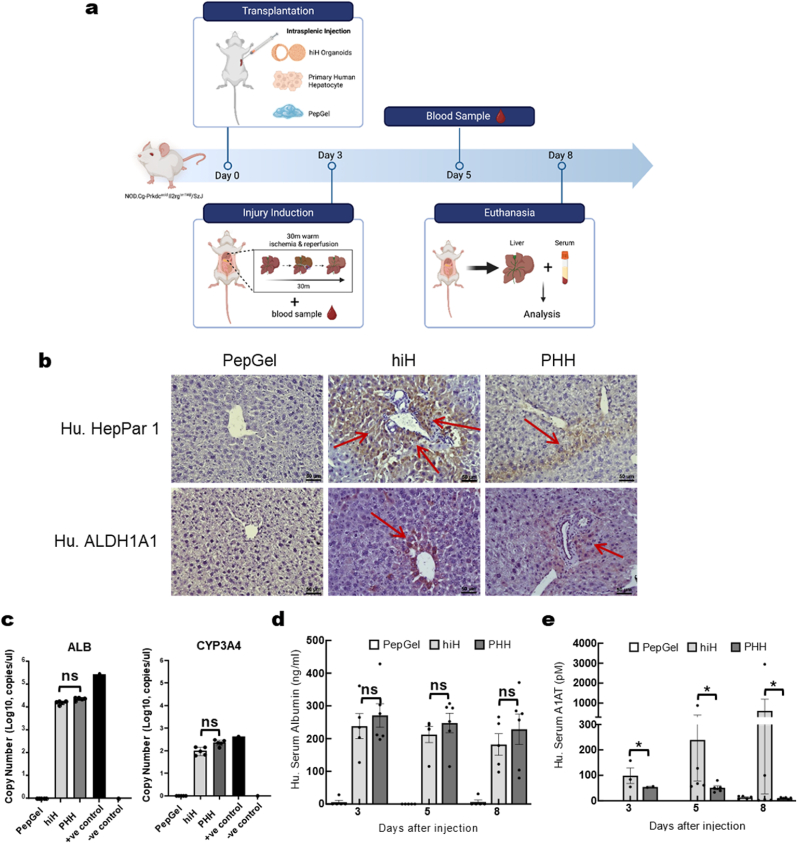
**An in vivo study of PG-hiHs revealed good engraftment into murine livers with comparable hepatocyte functional properties to those of PHHs. a,** Schematic illustration of the in vivo experimental design. **b,** Immunohistochemical images showing good engraftment of PG-hiHs into the murine liver (arrows) compared to that of Pepgel-only (negative control) and PHHs (positive control). Scale bar, 50 μm. **c,** Expression levels of human ALB and CYP3A4 mRNA in murine livers from mice injected with PG-hiHs or PHHs were not significantly different. The results are shown as the mean ± SEM (n = 5). **d,** Human serum ALB release by PG-hiHs was comparable to that by PHHs up to 8 days after injection. The results are shown as the mean ± SEM (n = 5). **e,** On average, PG-hiHs released more human serum A1AT than PHHs in vivo. The results are shown as mean ± SEM (n = 5). ns = not significant; ∗p < 0.05; ∗∗p < 0.01; ∗∗∗p < 0.001.

PG-hiHs differentiated from PG-sus were live-shipped overnight and then retrieved by centrifugation upon arrival. Within less than 6 h of receipt, PG-hiHs containing approximately 1x10^6^ hiH cells were delivered to the livers of up to 5 males, 8- to 12-week-old immunocompromised NSG (NOD. Cg-Prkdc^scid^Il2rg^tm1Wjl^/SzJ) mice via the splenic vein. Subsequently, hepatic ischemia‒reperfusion injury (IRI) was induced in the cephalad lobes of the mouse liver to induce a proliferative response (Fig. 5a). Livers retrieved on day 8 after injection were immunohistochemically stained for the presence of human-specific hepatocyte-specific antigen 1 (Hep Par 1) and aldehyde dehydrogenase 1 family member A1 (ALDH1A1). Both markers were readily detected in hepatocytes within the murine hepatic parenchyma (Fig. 5b). The expression levels of these genes in murine livers mirrored those observed in the livers of NSG mice 8 days after delivery of 1x10^6^ PHHs under identical experimental conditions (Fig. 5b). This finding suggested that live PG-hiHs retain their functionality after transplantation into immunocompromised mouse livers and can be efficiently integrated into these livers after IRI. These findings were corroborated by similar expression levels of human-specific *ALB* and *CYP3A4* mRNA in the murine livers of mice transplanted with both PG-hiHs and PHHs (Fig. 5c). Furthermore, we confirmed that these transplanted cells were functional as early as 3 days postinjection. Indeed, blood samples collected from mice at 3, 5 and 8 days post-transplantation showed a stable presence of human serum albumin, with concentrations comparable to those detected in mice transplanted with PHHs (Fig. 5d).

Similarly, human A1AT was detected in the blood of recipient mice (Fig. 5e). Interestingly, some mice transplanted with PG-hiHs exhibited significantly greater circulating levels of human-specific A1AT than did mice transplanted with PHHs. However, the results were not consistent across the entire study group, and observations over the study period were limited by sample availability. In contrast, none of these markers were detectable in the livers of the control mice injected with PepGel only. Overall, these data establish the successful engraftment of hepatocytes derived from PG-hiHs within the liver. This finding sets the stage for further research to validate these cells as potential sources for the development of autologous transplantation as a therapeutic approach for liver dysfunction.

### 3.6 Biofabrication of LC-hiPSC- and PG-hiH-laden constructs in PepGel-based bioink

In current tissue engineering studies, the viability and pluripotency of hiPSCs are often not well maintained during or after printing with bioinks [[64], [65], [66], [67], [68], [69]]. Our unique PepGel has been recently demonstrated for hiPSC 3D colonies bioprinting through extrusion with 96% cell viability and high pluripotency [28], which suggested potential for post-bioprinting differentiation. However, the bioprinted constructs by extrusion were limited to relatively simplistic shapes. Here, we designed and optimized PepGel-based multicomponent bioinks by blending them with PEGDA and employing the digital light processing (DLP) bioprinting technique to achieve high resolution bioprinting for more complex structures. DLP enables rapid, high-resolution, and layer-less fabrication of complex 3D architectures with excellent spatial fidelity and reproducibility, which are difficult to achieve using extrusion-based or inkjet bioprinting methods [[70], [71], [72]]. This was aimed at enhancing the printing resolution and structural complexity of the resulting constructs compared to those of the extrusion method, all while ensuring compatibility with LC-hiPSC 3D colonies.

**Fig. 6:**
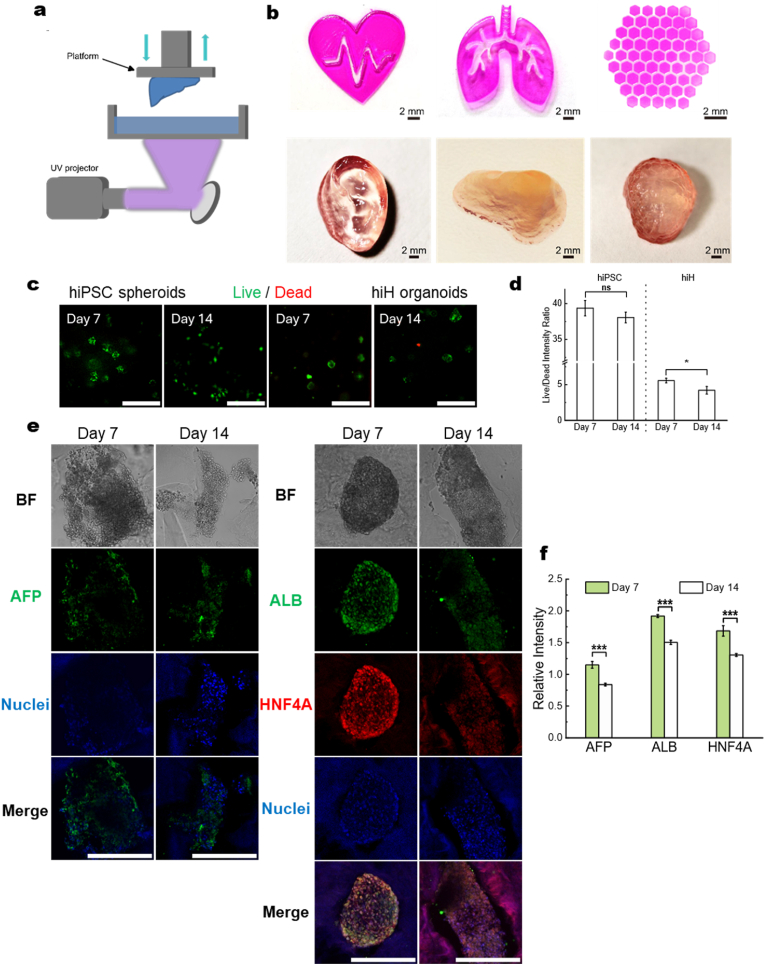
**DLP bioprinting with the optimized bioink allows the printing of 3D constructs with PG-hiHs while maintaining viability and hepatocyte characteristics for up to 14 days. a,** Illustration of the DLP bioprinter set up. **b,** The novel PepGel blended bioink can be used to create 2D and 3D constructs via DLP printing methods. Scale bar = 2 mm. **c,** Both hiPSC spheroids and PG-hiHs printed into PepGel blended bioink constructs maintained good viability after 7 and 14 days, as shown by live (green) and dead (red) viability staining. Scale bar, 500 μm **d,** Live and dead fluorescence intensity was quantified with Fiji-ImageJ, Live/Dead intensity ratio was shown as mean ± SEM (n = 3). **e,** PG-hiHs expressed the hepatocyte marker proteins AFP, ALB and HNF4A after they had been maintained for 7 and 14 days in bioprinted constructs. Scale bar, 200 μm. **f,** Quantification of AFP, ALB and HNF4A intensity from Fig. 6e, relative intensity = Target/DAPI, each image was measured 3 times, data shown as mean ± SEM (n = 3). ns = not significant; ∗p < 0.05; ∗∗p < 0.01; ∗∗∗p < 0.001.

A PepGel hybrid (PGS) was mixed with PEGDA at various ratios, after which the mechanical properties were characterized (Supplementary Figs. 10 and 11). The printed shapes were well maintained with 5% PEGDA +2% PGS (Supplementary Figs. 10a, b, 11a&b). In general, the compression modulus of PEGDA increased when 2-3% PGS was incorporated (Supplementary Figure 10c, 11c-e). As schematically illustrated in Fig. 6a, for DLP printing, the bioink was transferred to the vat of a typical DLP bioprinter, followed by crosslinking in a layer-by-layer manner as serial images were used to illuminate the vat. The optimized bioink containing 5% PEGDA and 2% PGS enabled the printing of diverse 2D tissue-like structures, such as the heart, lung, and liver lobules (Fig. 6b top). However, sophisticated 3D constructs, including those of the ear, nose, and brain, could be produced with our new bioink formulation (Fig. 6b bottom). When the bioink was laden with LC-hiPSC 3D colonies, live/dead staining (Fig. 6c, left two images) and fluorescence intensity quantification (Fig. 6d) conducted 7 and 14 days after printing, showed high and stable viability of the encapsulated LC-hiPSCs compared to those images taken on days 1 and 3 (Supplementary Fig. 12), which indicated favorable cytocompatibility of the bioink.

We proceeded to assess the printability of the PG-hiH 3D colonies using the new PEGDA/PGS (5%/2%) bioink. The printed constructs were then cultured in hepatocyte maintenance medium for 14 days. A live/dead assay conducted on days 7 and 14 revealed that the PG-hiHs within the constructs remained predominantly viable, as depicted in the right two images of Fig. 6c and the fluorescence intensity quantification (Fig. 6d). Furthermore, the expression of key liver functional biomarkers, *AFP*, *ALB*, and *HNF4A*, was detected and quantified in samples collected on both day 7 and day 14 (Fig. 6e and f), indicating that the liver function of the hepatocytes could be stably maintained within the bioprinted structures though slightly reduced by day 14.

## 4 Conclusion

This study establishes a fully defined and scalable synthetic peptide hydrogel platform that enables hiPSCs to self-organize into long-term, proliferative LC architectures and to undergo continuous, end-to-end differentiation into highly functional hepatocyte-like cells entirely within a 3D environment. By preserving intrinsic epithelial polarity and LC-driven self-organization throughout expansion and differentiation, this platform overcomes key limitations of conventional 2D and scaffold-free approaches that rely on stochastic aggregation and disrupt developmentally relevant tissue architecture.

LC-hiPSCs were routinely maintained for 3-20 passages in the PepGel 3D system prior to differentiation, and long-term genomic stability of this culture platform has been previously demonstrated by normal karyotype analysis over >40 passages [28]. To assess potential shipping-related stress, live-shipped LC-hiPSCs were evaluated post-delivery for viability, proliferation, and pluripotency marker expression, with no detectable loss of stemness.

The PepGel system integrates tunable mechanical properties, nanoscale fibrous structure, and cytocompatibility with bioprocessing-friendly features, enabling efficient LC-hiPSC expansion, differentiation, recovery, live shipment, and biofabrication without intermediate dissociation or animal-derived matrices. As a result, PepGel-derived hiPSC hepatocytes (PG-hiHs) form polarized, multi-luminal organoid structures and exhibit robust hepatic gene and protein expression, cytochrome P450 activity, and metabolic functions comparable to PHHs. Following transplantation, PG-hiHs engrafted efficiently and rapidly achieved human albumin and α-1 antitrypsin secretion at PHH-equivalent levels, demonstrating strong physiological relevance.

Beyond hepatic differentiation, this work introduces a generalizable strategy for organoid engineering by coupling intrinsic LC-mediated self-organization with a chemically defined synthetic ECM. The compatibility of both LC-hiPSCs and PG-hiHs with complex 3D bioprinted constructs further supports the utility of this platform for advanced tissue engineering and regenerative manufacturing.

Several limitations warrant further investigation. Long-term in vivo persistence, functional stability, and safety beyond early engraftment were not evaluated. In addition, while hepatocytes were selected as a representative endodermal lineage, broader validation across additional lineages and disease models remains to be explored.

Future studies will focus on extending long-term in vivo functional assessments, expanding lineage-specific applications, and integrating vascularization and multi-cellular complexity within bioprinted constructs. Together, these efforts are expected to further advance the translational potential of fully defined, bioactive hydrogel platforms for regenerative medicine and biofabrication.

## CRediT authorship contribution statement

**Quan Li:** Writing – review & editing, Writing – original draft, Visualization, Methodology, Investigation, Formal analysis, Data curation. **Guangyan Qi:** Writing – review & editing, Writing – original draft, Visualization, Methodology, Investigation, Formal analysis, Data curation. **Ling Cai:** Writing – review & editing, Visualization, Methodology, Investigation, Formal analysis, Data curation. **Abhishek P. Shrestha:** Writing – review & editing, Visualization, Methodology, Investigation, Formal analysis, Data curation. **Sergio Duarte:** Writing – review & editing, Visualization, Validation, Supervision, Methodology, Investigation, Formal analysis, Data curation. **Yu Shrike Zhang:** Writing – review & editing, Visualization, Supervision, Project administration, Methodology, Investigation, Funding acquisition, Conceptualization. **Ali Zarrinpar:** Writing – review & editing, Visualization, Supervision, Project administration, Investigation, Funding acquisition, Conceptualization. **Tawfik Aboellail:** Writing – review & editing, Visualization, Methodology, Investigation, Formal analysis, Data curation. **Xuming Liu:** Writing – review & editing, Methodology, Data curation. **Jianfa Bai:** Writing – review & editing, Supervision, Resources, Methodology, Data curation. **Md Sharifur Rahman:** Writing – review & editing, Methodology, Formal analysis, Data curation. **Bruno Hagenbuch:** Writing – review & editing, Validation, Methodology, Data curation. **Ming-Shun Chen:** Writing – review & editing, Resources, Methodology. **Anthony Atala:** Writing – review & editing, Supervision, Resources, Funding acquisition. **Xiuzhi Susan Sun:** Writing – review & editing, Writing – original draft, Visualization, Validation, Supervision, Resources, Project administration, Methodology, Investigation, Funding acquisition, Conceptualization.

## Ethics approval and consent to participate

All animal studies and procedures were conducted in accordance with the ethical guidelines established by the National Institutes of Health animal care and use and were approved by the University's Animal Care and Use Committee (201709890). This article does not contain any studies with human subjects performed by any of the authors.

## Declaration of competing interest

YSZ consulted for Allevi by 3D Systems; consults for PepGel; cofounded, consults for, and holds options of Linton Lifesciences; cofounded, consults for, and holds options of Criocore; and sits on the scientific advisory board and holds options of Xellar Biosystems. The relevant interests are managed by the Brigham and Women's Hospital.

XSS founded PepGel, LLC and served as the chief technology officer.

The other authors declare no competing interests.
