# Supplementary material for: Scalable synthetic peptide hydrogel enables self-organized luminal cavity architecture within hiPSC 3D colonies supporting functional hepatocyte differentiation
Source: Bioact Mater. 2026 Apr 1;62:814–30. doi: 10.1016/j.bioactmat.2026.03.029 (PMC13084690; doi:10.1016/j.bioactmat.2026.03.029)
Supplement: Multimedia component 1 [file mmc1.docx]

**In the Supporting Information, there was a duplication of Table 5 due to an error. One of these tables has now been renumbered as Table 6, as shown below, and the corresponding citation has been updated in the main manuscript. Please renumber Table 5 to Table 6 in the Supporting Information, following the revised PDF sent by email dated March 18, 2026.**

**Supplementary Table 5.** LC-hiPSC–derived hepatic organoids (PG-hiHs) cultured in PGmatrix suspension (PG-sus) exhibited significantly higher differentiation efficiency than hepatic aggregates cultured in U96 wells.

| Sample | Albumin (%) | HNF4A (%) |
| --- | --- | --- |
| Control hiPSC | 26.30±3.2^c^ | 0.00±0.00^c^ |
| Hepatic aggregate in U96 | 77.80±6.3^b^ | 30.8±2.6^b^ |
| PG-hiH in 3D PG-sus | 94.8±3.9^a^ | 39.6±3.1^a^ |

**Supplementary Table 6.** LC-hiPSC–derived hepatic organoids (PG-hiHs) cultured in PGmatrix suspension (PG-sus) exhibited significantly higher differentiation efficiency than hepatic aggregates cultured in U96 wells.

| Sample | Albumin (%) | HNF4A (%) |
| --- | --- | --- |
| Control hiPSC | 26.30±3.2^c^ | 0.00±0.00^c^ |
| Hepatic aggregate in U96 | 77.80±6.3^b^ | 30.8±2.6^b^ |
| PG-hiH in 3D PG-sus | 94.8±3.9^a^ | 39.6±3.1^a^ |
