# Supplementary material for: Scalable synthetic peptide hydrogel enables self-organized luminal cavity architecture within hiPSC 3D colonies supporting functional hepatocyte differentiation
Source: Bioact Mater. 2026 Apr 1;62:814–30. doi: 10.1016/j.bioactmat.2026.03.029 (PMC13084690; doi:10.1016/j.bioactmat.2026.03.029)
Supplement: Multimedia component 2 [file mmc2.pdf]

Supporting Information

1. Supporting Results and Figures

1.1. Fluorescence morphologies of 3D luminal cyst (cavity) structured hiPSC (LC-hiPSC) colonies in both PG-embedded and PG-sus and hiPSC aggregates stained by selected protein markers (Supplementary Figure 1a, 1b, 1c).

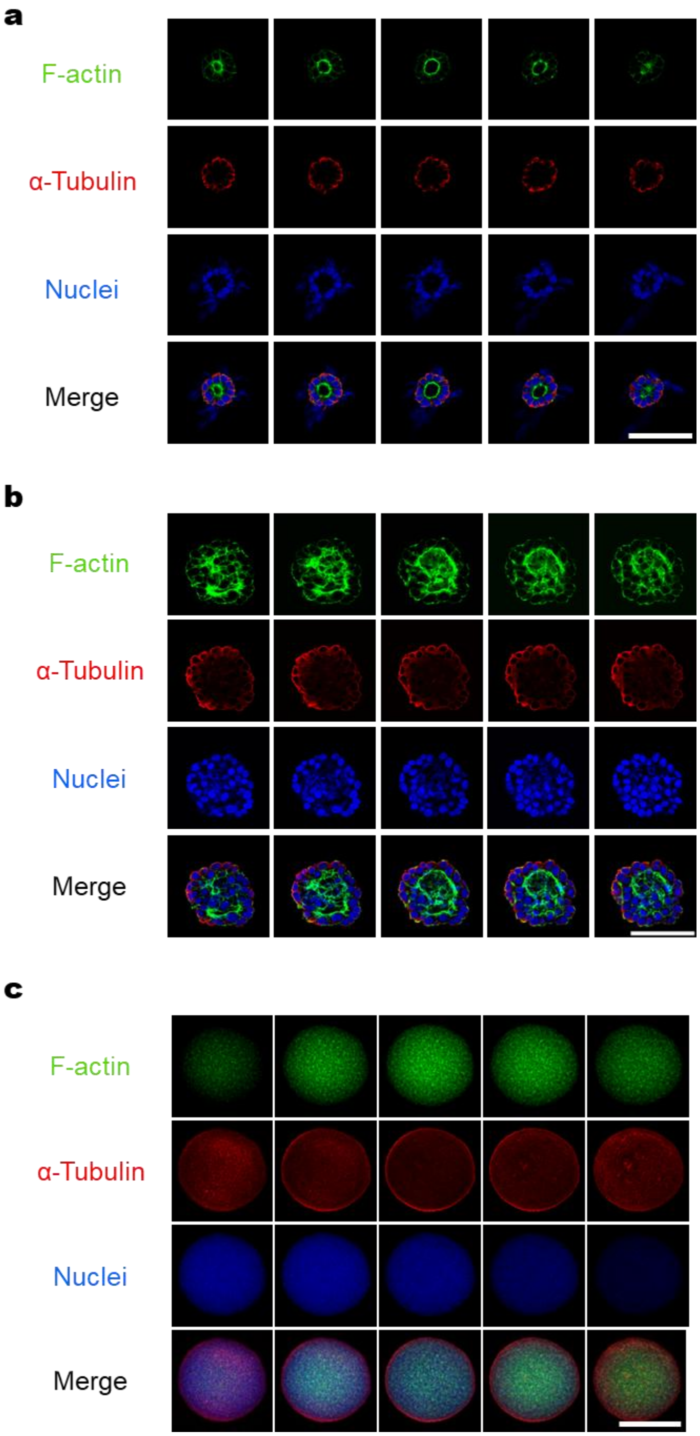

d e

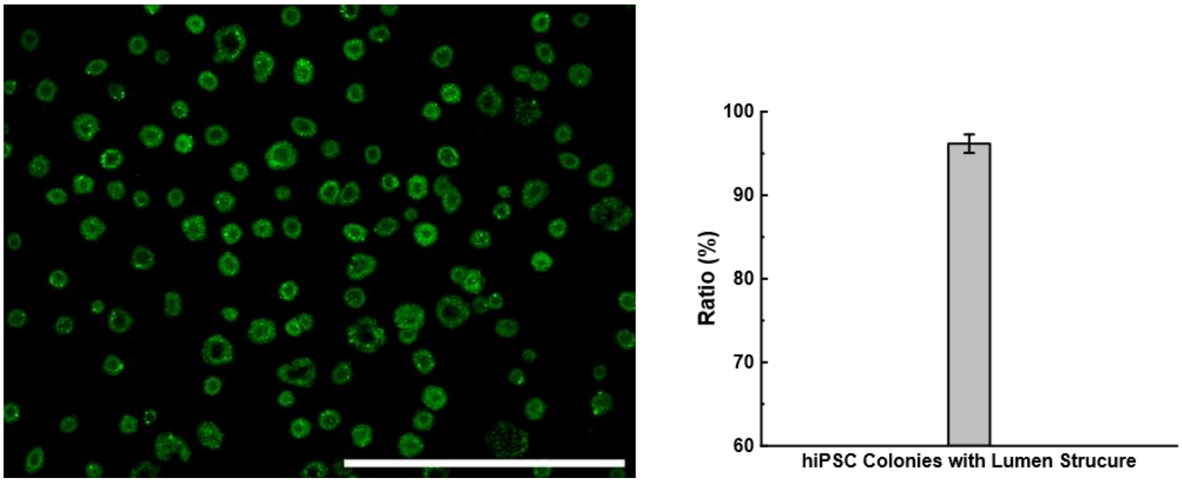

Supplementary Figure 1. Z-stack images revealed patterned F-actin and α-Tubulin staining for 3D LC-hiPSCs compared to hiPSC aggregates. Z-stack images of F-actin-, α-Tubulin-, and Nuclei-stained 3D LC-hiPSC colonies from **a**, PG-embedded, scale bar 200µm and **b**, PG-sus, scale bar 200 µm. **c.** Z-stack images of F-actin-, α-Tubulin-, and Nuclei-stained hiPSC Aggregates from non-adherent U-bottom 96 well suspension, scale bar 600µm. **d.** morphologies of hiPSCs, as an example, used for quantifying the percentage ratio of 3D hiPSC colonies with lumen structure in them, scale bar 1000µm. **e.** Ratio of 3D hiPSCs with lumen cyst structure obtained from image quantification. Data shown as mean ± SEM (n=4). Method for lumen quantification was described in section 2.1 of this Supplementary document.

1.2. Luminal cyst morphogenesis of hiPSCs 3D colonies were severely disrupted in 3D culture conditions by either culture medium, synthetic ECM (e.g.,Vitronectin), or natural ECM (e.g., Matrigel derived from mouse tumors) (Supplementary Figures 2, 3, 4)

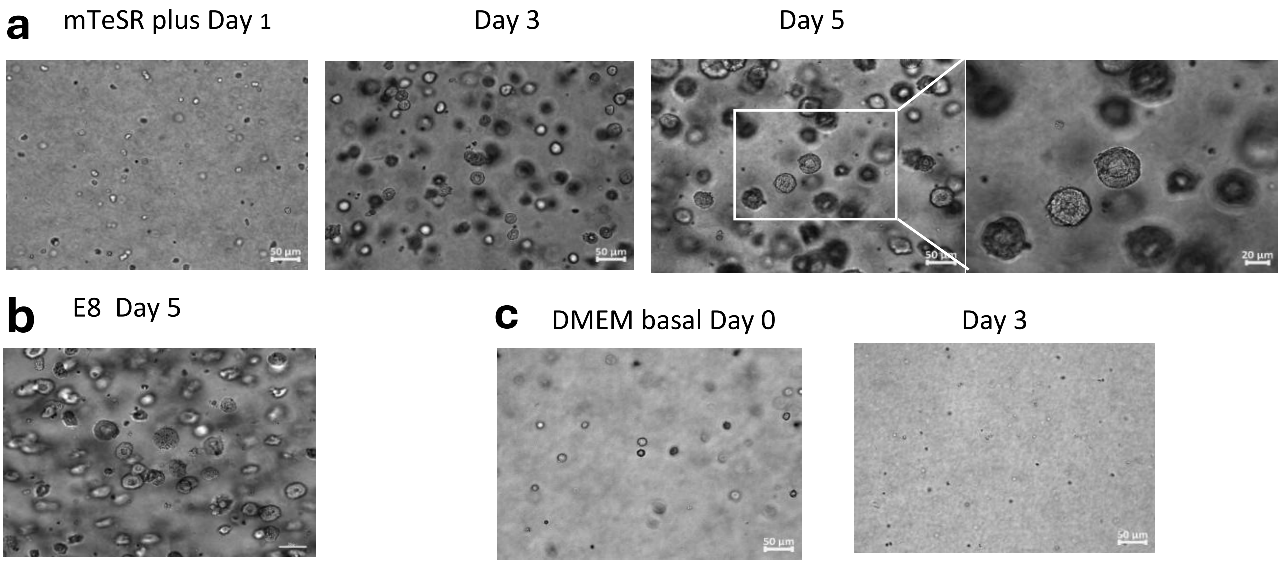

Supplementary Figure 2. Morphogenesis of 3D LC-hiPSC colonies is influenced significantly by growth factors presented in the medium. hiPSCs were cultured in PGmatrix (PGmatrix 3D hiPSC Kit, PepGel, LLC, Winston Salem, NC USA) supplemented with ROCK inhibitor Y27632 using **a.** mTeSR plus complete medium; **b.** Essential 8 (E8) medium and **c.** DMEM basal medium.

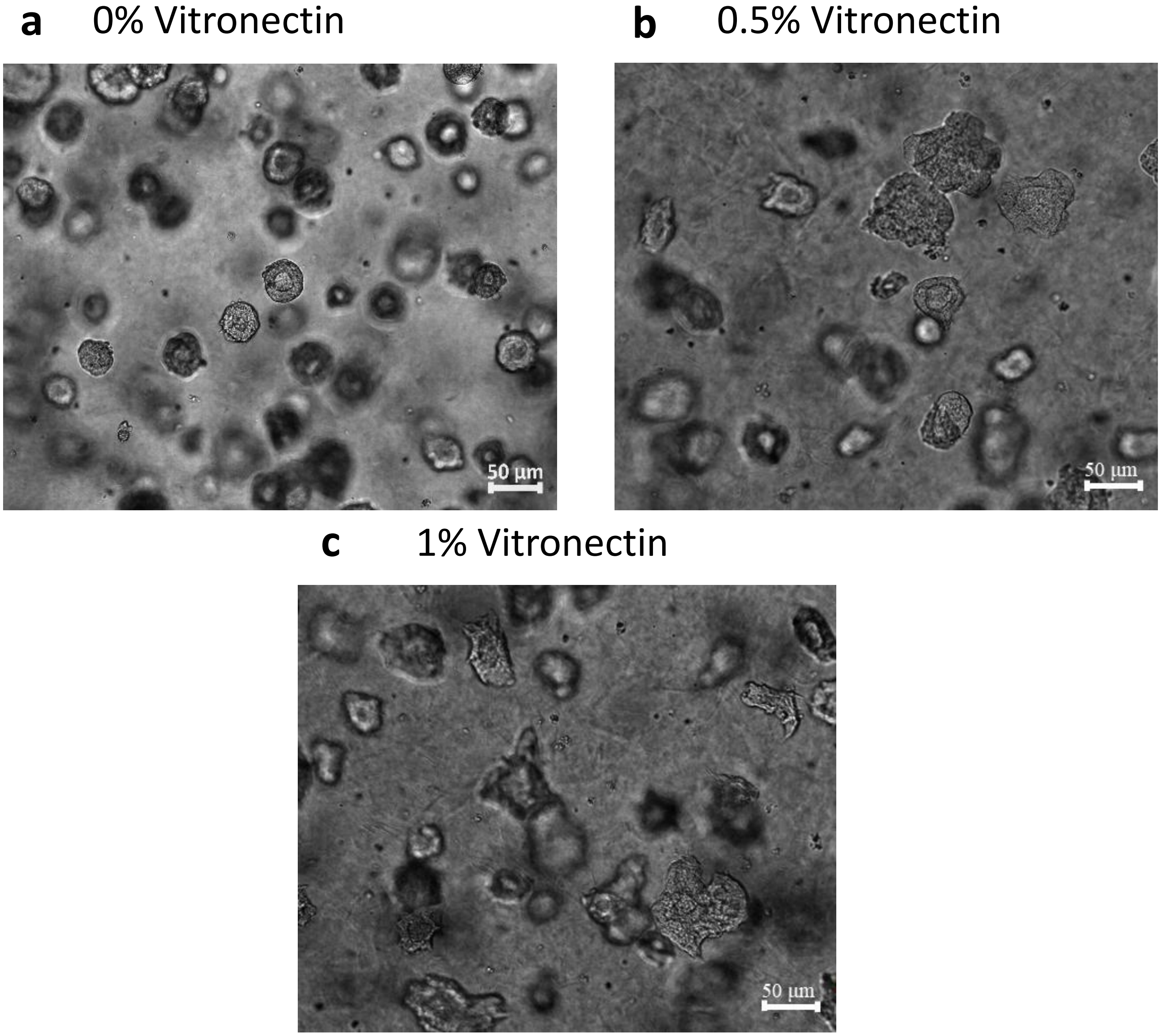

Supplementary Figure 3. Morphogenesis of 3D LC-hiPSC colonies is influenced significantly by scaffold composition, such as synthetic ECM Vitronectin. hiPSCs were cultured in PGmatrix 3D hiPSC with varied Vitronectin protein for 5 days using mTeSR plus complete medium supplemented with ROCK inhibitor Y27632. **a.** 0% Vitronectin on day 5; **b.** 0.5% Vitronectin on day 5; **c.** 1% Vitronectin on day 5.

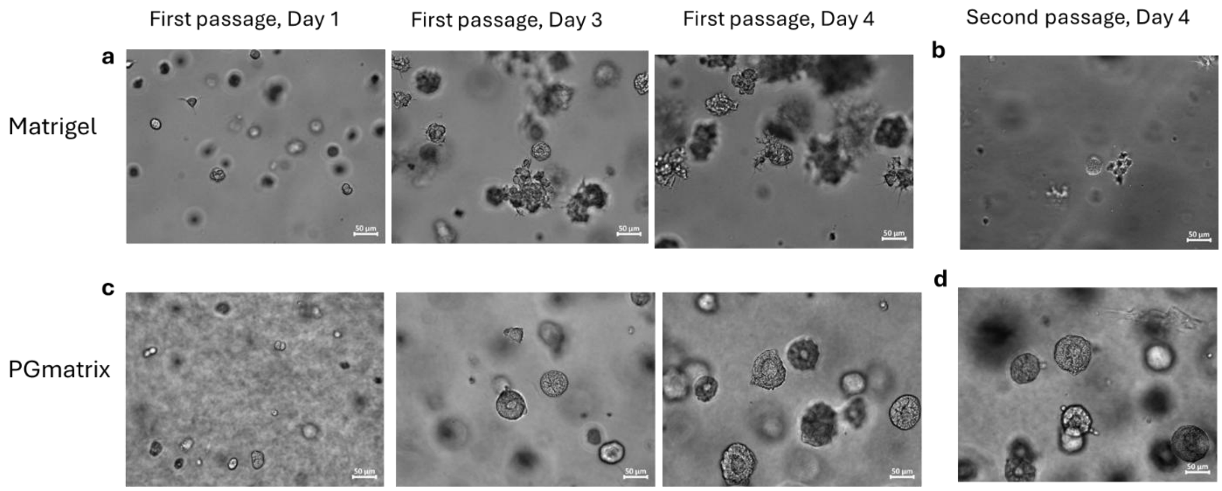

Supplementary Figure 4. **Morphogenesis of 3D LC-hiPSC colonies is severely disrupted by Matrigel.** hiPSCs were cultured in 3D for 4 days using mTeSR plus complete medium supplemented with ROCK inhibitor Y27632. hiPSCs morphologies in **a.** Matrigel passage 1; **b.** Matrigel passage 2; **c.** PGmatrix passage 1; and **d.** PGmatrix passage 2.

1.3. Pilot study of hepatocyte differentiation: protocol validation and biomarkers

We initially evaluated the differentiation protocol across three distinct 3D environments to optimize conditions for hepatic differentiation. LC-hiPSC 3D colonies were either encapsulated in PGmatrix embedded (PG-embedded)[4] or plated onto wells coated with PGmatrix (PG-coating) as suspension. Additionally, hiPSC single cells were plated in ultra-low attachment U-96 well plates to aggregate into spheroids, mimicking suspension-based differentiation. These groups were compared with primary human hepatocytes (PHHs) serving as the positive control.

Differentiation stage biomarkers: The modified differentiation protocol incorporated an additional stage to direct the development of foregut endoderm towards hepatoblast cells, thereby minimizing potential differentiation towards cholangiocytes[5]. Following the completion of each differentiation stage, the hepatocyte 3D colonies were retrieved by centrifugation, and the expression of key genes was characterized by reverse transcription quantitative polymerase chain reaction (RT-qPCR) (**Supplementary Figure 5**). hiPSC aggregates were differentiated following the same protocol and were matured in Matrigel following the method by Pettinato[5], while primary human hepatocytes (PHH) served as positive control for gene marker analysis.

As differentiation progressed, both PG-coating and PG-embedded exhibited elevated expressions of differentiation marker genes overall (**Supplementary Figure 5**). By the end of stage 4, both

PG-embedded and PG-coating outperformed the hiPSC aggregates generated from widely used scaffold-free suspension systems. The hepatocyte 3D colonies from the PGmatrix system showed significantly higher expression levels of key functional hepatocyte genes, including α-fetoprotein (AFP), albumin (ALB), hepatocyte nuclear factor 4 alpha (HNF4A), and transthyretin (TTR) (**Supplementary Figure 5d**). Furthermore, the PG-coating hepatocyte colonies also expressed significantly higher levels of α1-antitrypsin (A1AT), asialoglycoprotein receptor 1 (ASGR1), cytochrome P450 3A4 (CYP3A4), tryptophan 2,3-dioxygenase (TDO2), and transthyretin (TTR) (**Supplementary Figure 1d**). However, the hepatocytes in PG-coating formed giant organoids due to fusion because of their adherent nature and precipitation (**Supplementary Figure 5e**).

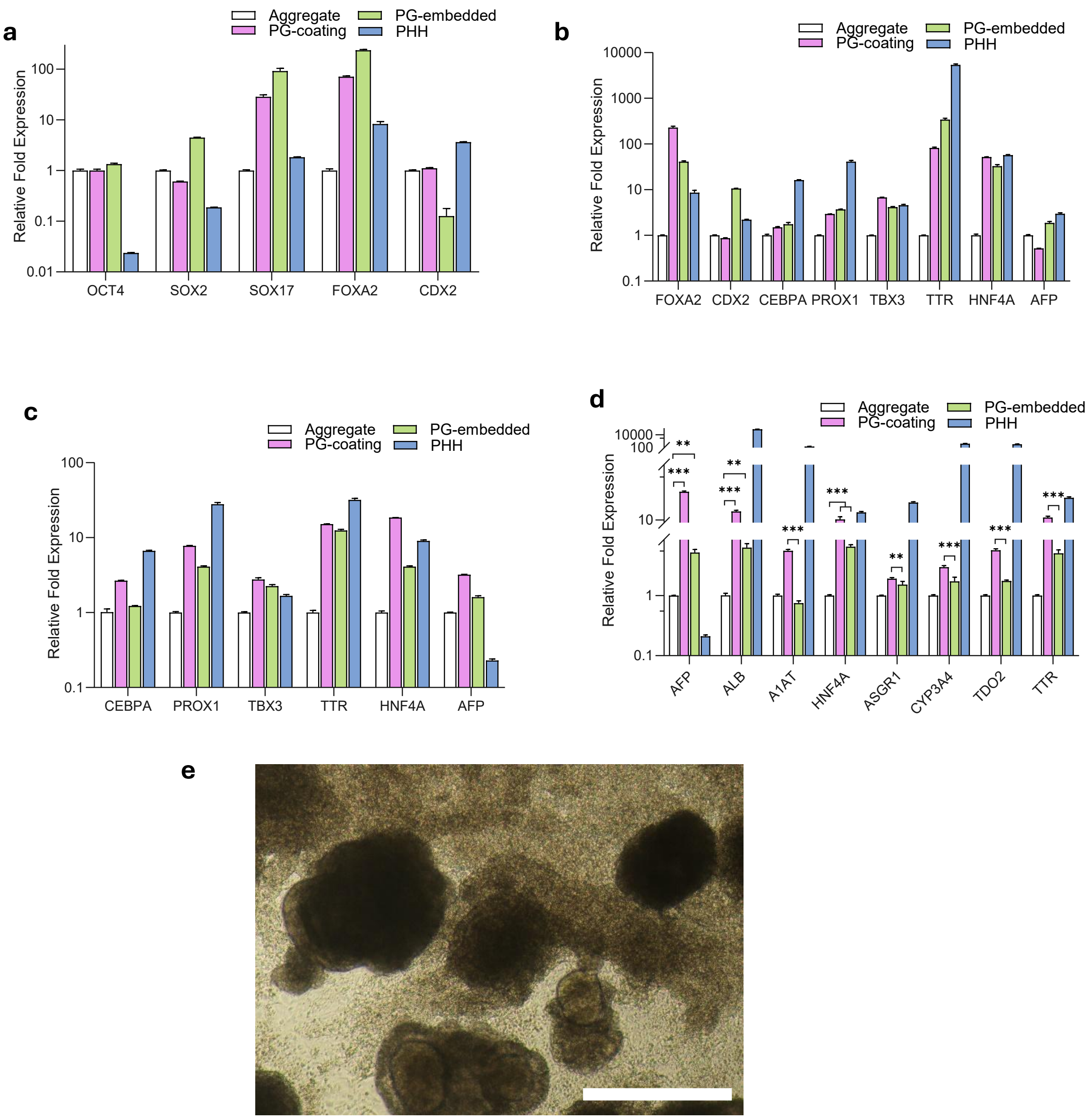

Supplementary Figure 5. Validation of Hepatocyte differentiation. PCR results of hepatocyte organoids differentiated in various 3D environments to validate the 3D differentiation protocol. **a.** PCR analysis of marker genes for stage 1 Definitive endoderm, **b.** stage 2 Foregut endoderm, **c.** stage 3 Hepatoblasts, **d.** end of differentiation at stage 4 Hepatocyte-like cells; results shown as mean ± SEM (n=3), *$p < 0.05$; **$p < 0.01$; ***$p < 0.001$. **e.** Morphology of differentiated spheroids in PG-coating on Day 12. Scale bar 300μm.

1.4.Differentiation efficiency of hiPSCs in PG-sus and Aggregate using Flow Cytometry method. Hepatic-differentiated hiPSCs cultured in PG-sus exhibited markedly higher marker expression than those in U96 aggregates: 95% vs 78% albumin-positive cells and 40% vs 31% HNF4A (HNF--4α)–positive cells, respectively (**Supplementary Table 5**).

a

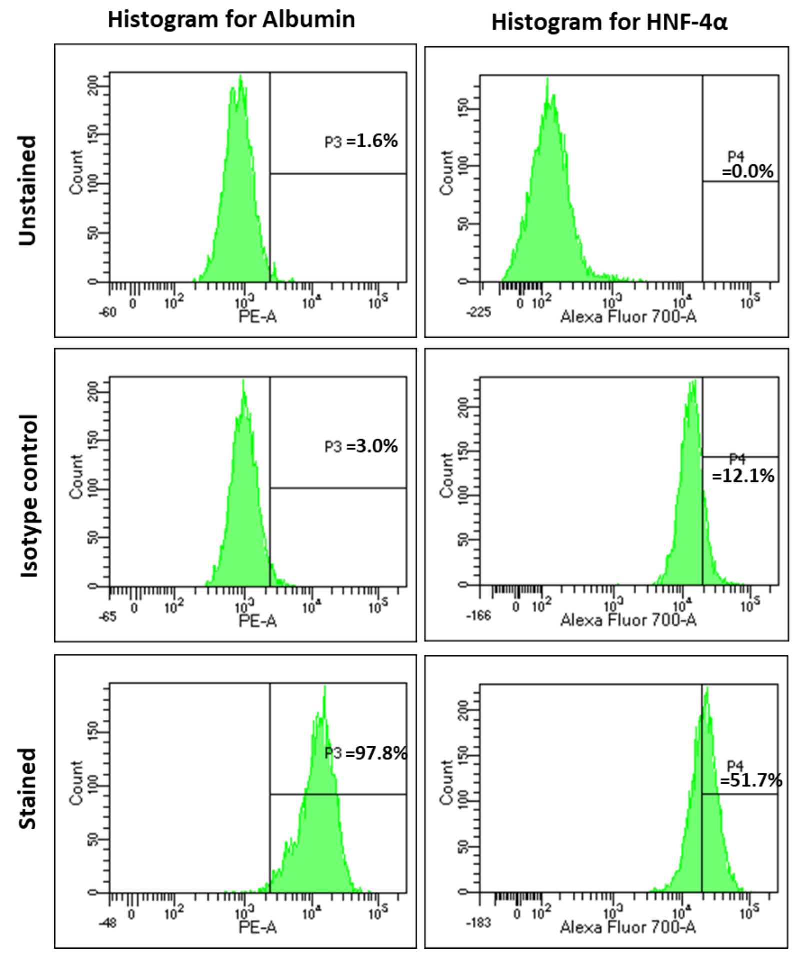

b

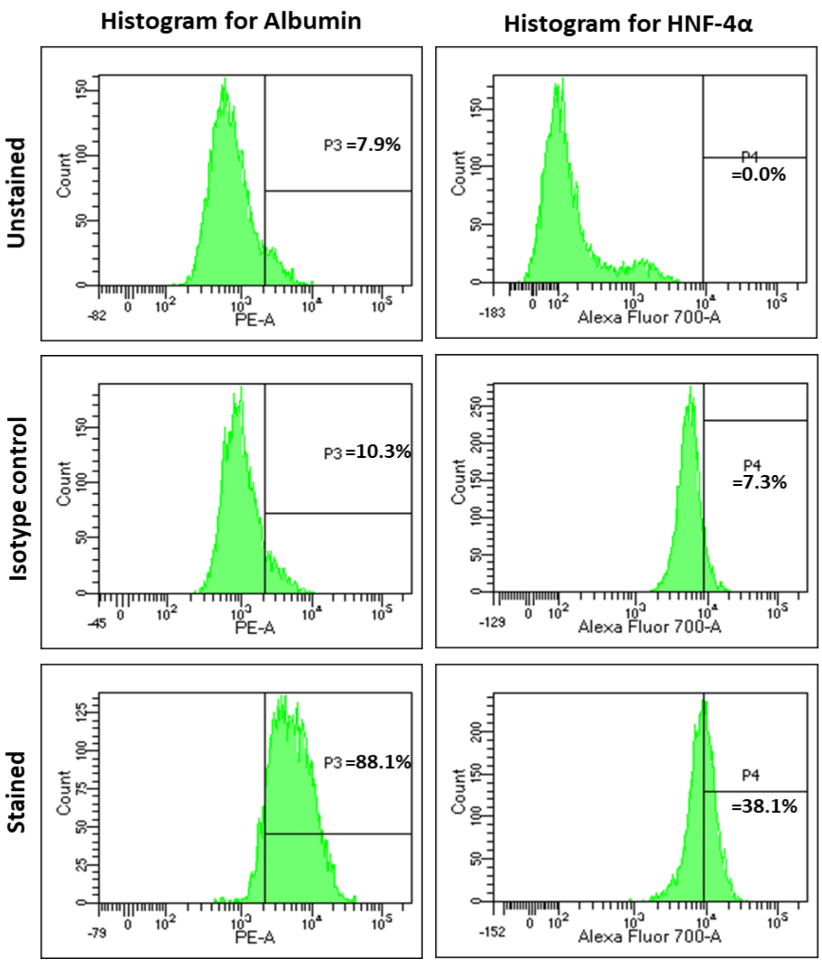
Histogram for Albumin
Histogram for HNF-4α
Unstained
Isotype control
Stained
Count
PE-A
Alexa Fluor 700-A
P3 =7.9%
P4 =0.0%
P3 =10.3%
P4 =7.3%
P3 =88.1%
P4 =38.1%

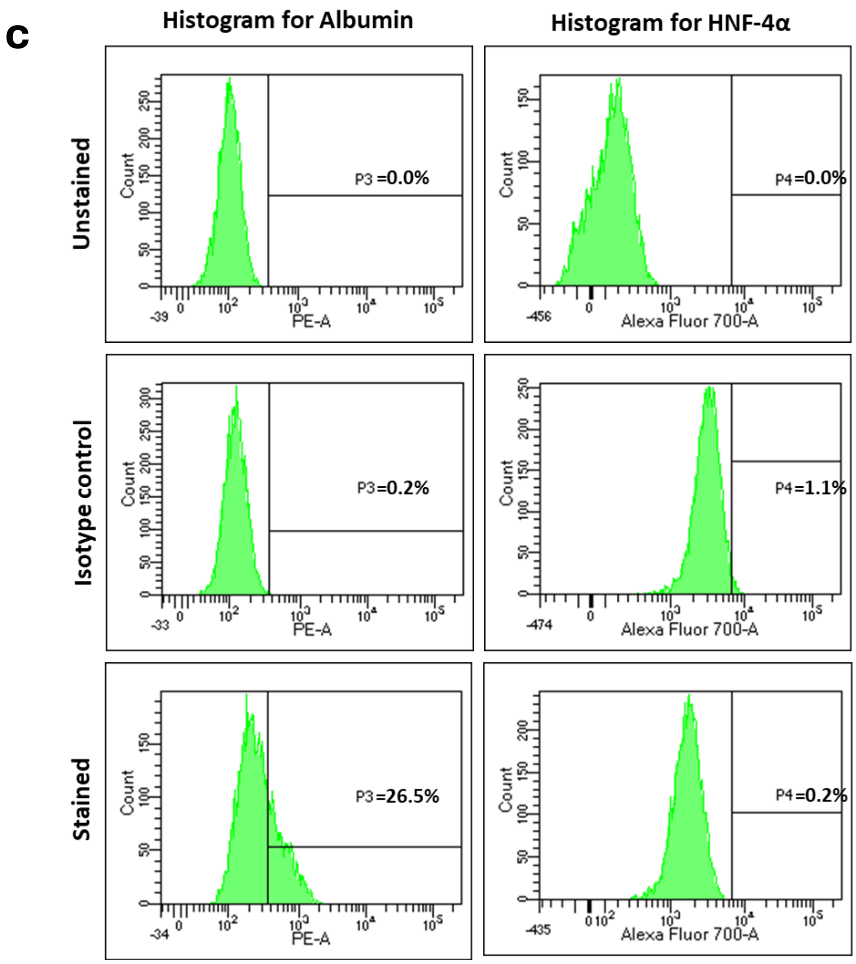

Supplementary Figure 6. Flow cytometry analysis showed that differentiation efficiency of PG-hiH is significantly higher than that of Aggregate. a. PG-hiHs differentiated in PG-sus; b. hepatic Aggregates differentiated in U96 well plate; c. undifferentiated hiPSCs for comparison purposes.

1.5 [renumber after this] Distribution of hepatocyte marker proteins was different on PG-sus and Aggregate hiHs.

As shown in **Supplementary Figure 7** and **Supplementary Videos 1&2**, hepatocyte marker proteins ALB and HNF4A were uniformly distributed within PG-hiHs, with fluorescence from stained proteins appearing bright across various Z planes. In contrast, in Aggregate spheroids, fluorescence from ALB and HNF4A was predominantly bright on the surface, gradually dimming as it approached the inner center of the hiPSC derived hepatocyte aggregate.

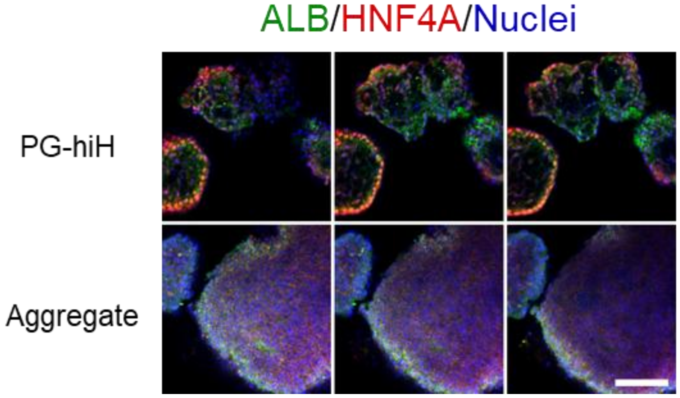

Supplementary Figure 7. Z-stack images of sliced hiPSC-derived PG-hiH and Aggregate stained for polarity marker ABCB11 and α1 Sodium Potassium ATPase from surface (left) to center (right). Scale bar 200µm.

Supplementary Video 1. Z-stack video of PG-hiH spheroids stained for ALB (Green), HNF4A (Red) and the nuclei (Blue).

Supplementary Video 2. Z-stack video of differentiated aggregate, generated using scaffold free method, stained for ALB (Green), HNF4A (Red) and the nuclei (Blue).

1.6. Quantification of staining intensity of mature hepatocytes expressing hepatocyte paraffin 1 (Hep Par 1) and proliferation marker (Ki-67).

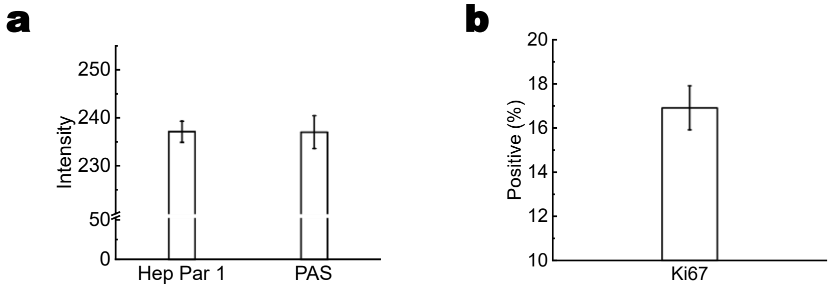

Supplementary Figure 8. Quantification of staining intensity. a, Hep Par 1 (Figure. 3g) and PAS (Figure 3h), data shown as mean ± SEM (n=4 quadrants). **b,** Percentage of cells positive for Ki67 was quantified and shown in bar graph as mean ± SEM (n=3).

1.7 Live shipping of cell spheroids in PepGel hybrid hydrogel can preserve hiPSC viability and pluripotency.

We initially assessed the live shipping using PepGel hybrid PGmatrix-LiveShip Kit (PG-LS) (PepGel LLC) for 3D LC-hiPSCs, given their susceptibility to environmental changes. LC-hiPSCs were encapsulated in PG-LS and placed at room temperature in duplicate overnight, followed by retrieval via centrifugation. Across all batches tested, the viability of hiPSCs consistently exceeded 96% (**Supplementary Figure 9a**). Subsequently, we conducted live shipping of LC-hiPSCs to Applied Stemcell Inc (ASC) in California under the temperature range -10°C to 20°C. Upon arrival, the LC-hiPSCs were retrieved and cultured in 2D for one passage before undergoing tri-lineage differentiation to verify pluripotency. Comparative analysis with locally cultured hiPSCs (Ctrl) revealed no significant differences in morphology or proliferation ability for the live shipped LC-hiPSCs (**Supplementary Figure 9b**). Furthermore, tri-lineage differentiation assays demonstrated that these live shipped LC-hiPSCs retained their pluripotent capabilities (**Supplementary Figure 9c**, **d, e**). Collectively, these findings indicate that live shipping effectively preserves cell viability and functional properties.

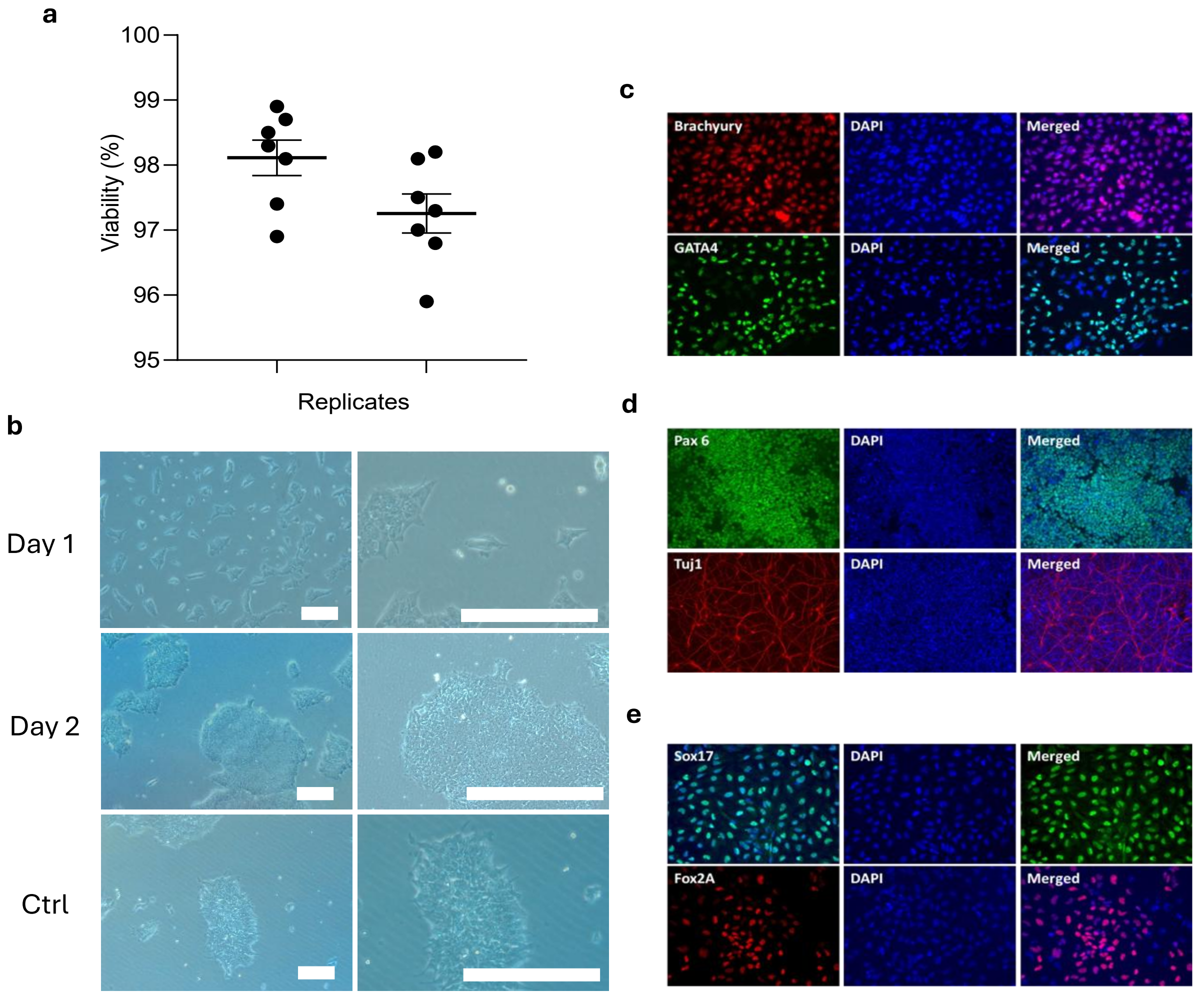

Supplementary Figure 9. Results from shipping test with LC-hiPSCs showed excellent viability and proliferation, as well as pluripotency maintenance after live-shipping at ambience temperature. The live-shipping method was developed by encapsulating the LC-hiPSC spheroids in PG-LS and placing them in a conical centrifuge tube with culture medium on top, which can be maintained and shipped at ambient temperature. **a.** Cell viability of dissociated LC-hiPSCs measured upon receipt of live shipped LC-hiPSC spheroids in different batches. Results shown as mean ± SEM. **b.** Morphology of lived shipped hiPSCs cultured in 2D on Day 1 and Day 2 in comparison with Day 2 locally cultured hiPSCs (Ctrl) at low (left) and high (right) magnification. Scale bar 150µm. **c-e.** The dissociated live shipped LC-hiPSC were cultured in 2D then subjected to trilineage differentiation. After differentiation, the cells showed expression of marker proteins for all three lineages: mesoderm (c), ectoderm (d), and endoderm (e).

1.8. Bioink formulation

PepGel hybrid (PGS) (PGmatrix Spheroids Kit (PepGel LLC)) was mixed with polyethylene glycol diacrylate (PEGDA) at various ratios and characterized for mechanical properties and cytocompatibility. Viscosity is a crucial parameter for bioink solution in terms of cell encapsulation efficiency and printing resolution. The viscosity of PEGDA-PGS composites regardless of mixing ratios mainly remained the same as that of PEGDA alone (**Supplementary Figure 10a**), indicating printing parameters for PEGDA-PGS should be similar as printing PEGDA alone. The PEGDA-PGS bioinks, containing photoinitiator lithium phenyl-2,4,6-trimethylbenzoylphosphinate (LAP), were UV-crosslinked after pipetting into a disk mold (8 mm × 1 mm) at 80 mW for 35 s and the cured samples were further tested for mechanical properties (**Supplementary Figure 10b**). In general, the compression module of PEGDA significantly increased by incorporating 2-3% of PGS (**Supplementary Figure 10c**). It could be attributed to that the nanofibers of PGbioink acted as the reinforcing nanofillers within the PEGDA matrix, filling the space between polymer chains to form a denser network, which strengthened the 3D network leading to enhanced mechanical performances. For example, the pure 1.5% PEGDA solution under the same UV curing condition was hard to form a disk, while by incorporating 3% PGS, the well-defined, self-supporting disk can be readily obtained (**Supplementary Figure 10b**), which had the compression storage modulus of 6 kPa (**Supplementary Figure 10c**). At 5% PEGDA, the storage modulus increased up to 24 kPa with the addition of 2% PGbioink, compared to 16 kPa of pure PEGDA (**Supplementary Figure 10c**). Accordingly, we evaluated the printability of these bioink formulations with DLP and observed the same trend; at 1.5% PEGDA even with 3% PGS added, the shape fidelity of the rectangular sample produced was inferior, while the 5% PEGDA would support printing of well-structured cube at 2% PGS.

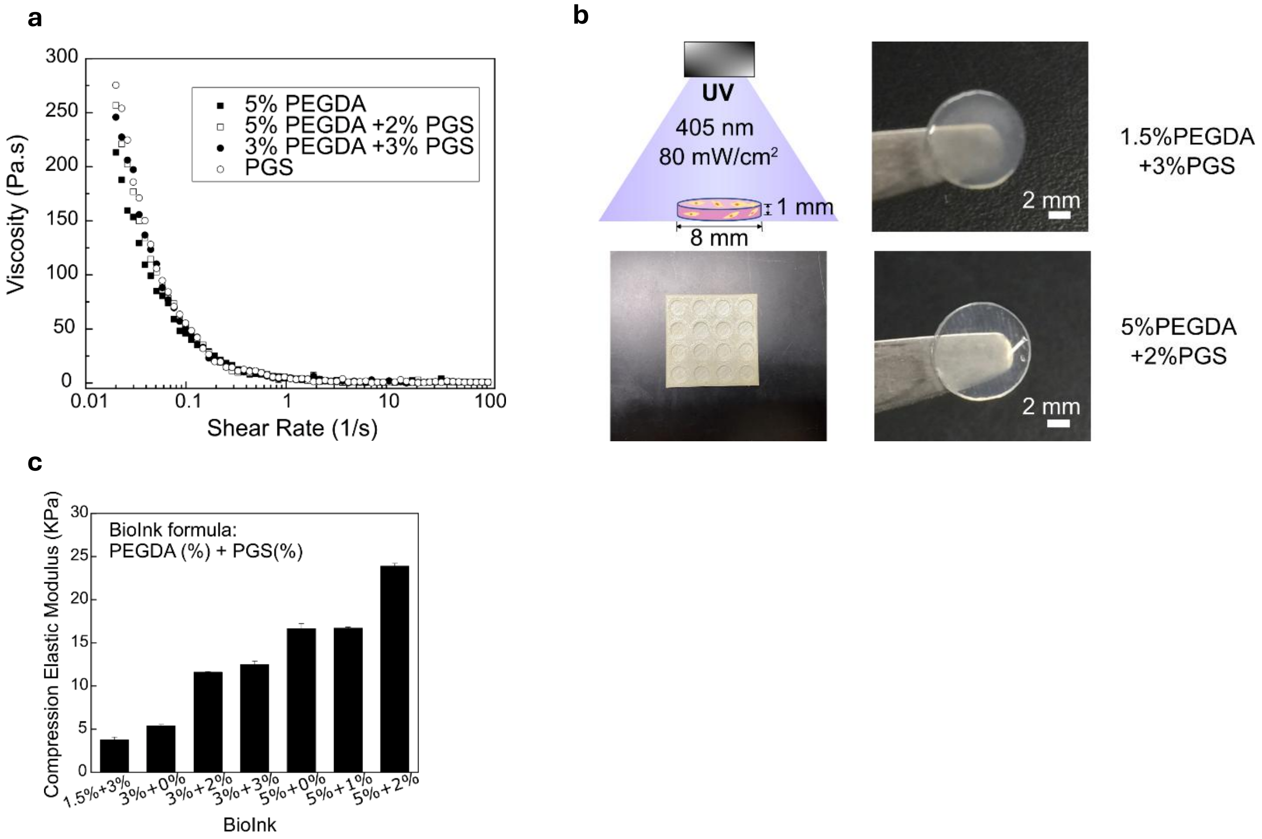

Supplementary Figure 10. Formulation of PEGDA-PGbioink (PGS) blend bioink. **a.** PGS was mixed with PEGDA at various ratios and characterized for mechanical properties and cytocompatibility. The viscosity of PEGDA-PGS composites regardless blending ratios mainly remained the same as that of PEGDA alone. **b.** PEGDA-PGS bioinks, containing photoinitiator lithium phenyl-2,4,6-trimethylbenzoylphosphinate (LAP), were UV-crosslinked after pipetting into a disk mold (8 mm × 1 mm) at 80 mW/cm^2 for 35s to produce disk-shaped samples. Scale bar 2mm. **c.** The compression moduli of PEGDA increased by incorporating 2-3% of PGS. Results shown as mean ± Standard deviation (STD).

1.9. DLP bioprinting of PEGDA-PGbioink (PGS) composites

To assess the mechanical properties of PEGDA-PGS bioinks, rectangular samples were fabricated by DLP printing for additional tensile and compression tests using bioinks containing either 1.5% or 5% PEGDA in combination with 3% or 2% PGbioink, respectively (**Supplementary Figure 11a, b**). Tensile test results indicated that the constructs comprising 5% PEGDA and 2% PGS achieved a tensile strain of 60% (**Supplementary Figure 11c**). In contrast, the constructs composed of 1.5% PEGDA and 3% PGS could not even be subjected to testing due to insufficient support from the PEGDA polymer network to form a cohesive structure, as shown in **Supplementary Figure 11a, b**.

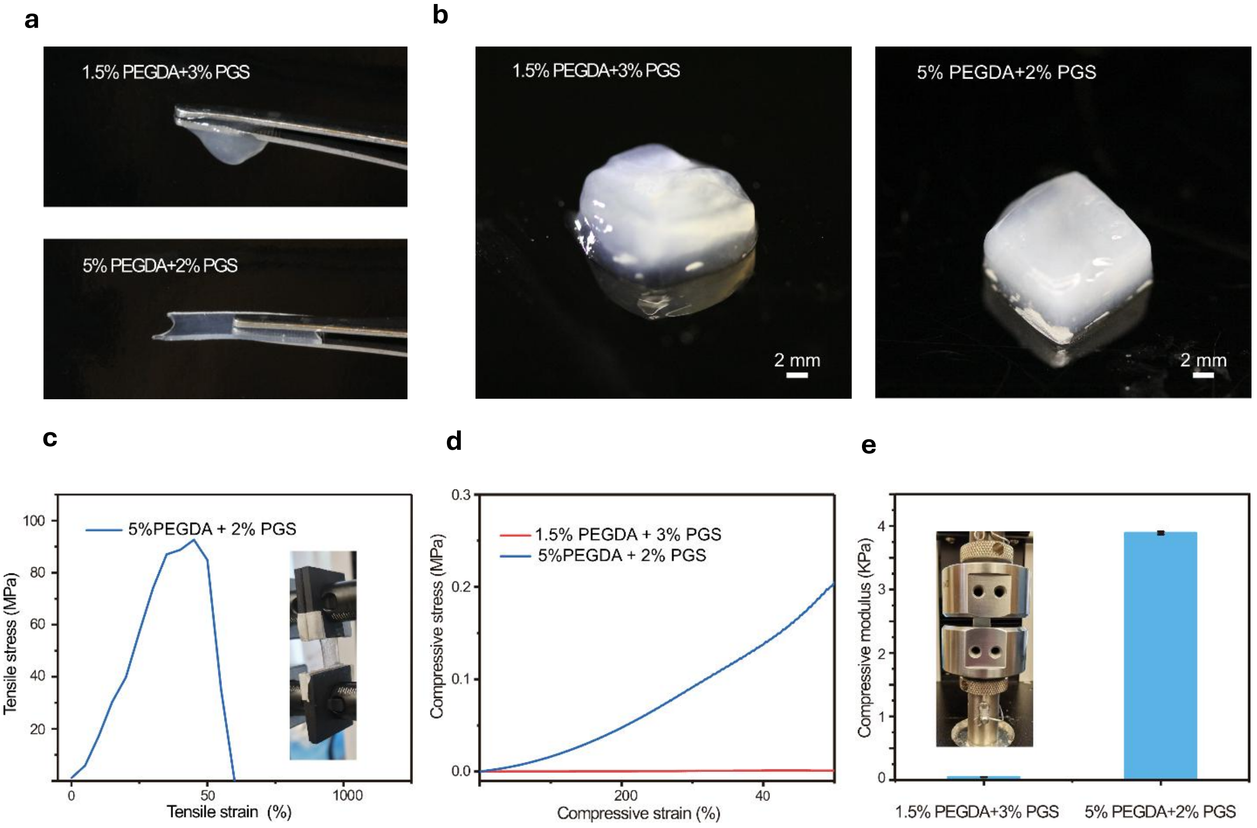

Supplementary Figure 11. Constructs produced from PEGDA-PGS bioinks through DLP printing method were analyzed for mechanical properties. **a&b.** Rectangular samples were fabricated by DLP printing using PEGDA-PGS bioinks containing either 1.5% or 5% PEGDA in combination with 3% or 2% PGS. Scale bar 2mm. **c.** Tensile test results indicated that the constructs comprising 5% PEGDA and 2% PGS achieved a tensile strain of 60%. **d.** Compressive strain of bioinks at different blending ratio. **e.** Compressive moduli of bioinks at different blending ratio.

1.10.. Live/Dead staining images of post-bioprinted 3D LC-hiPSCs after DLP bioprinting (Supplementary Figure 12).

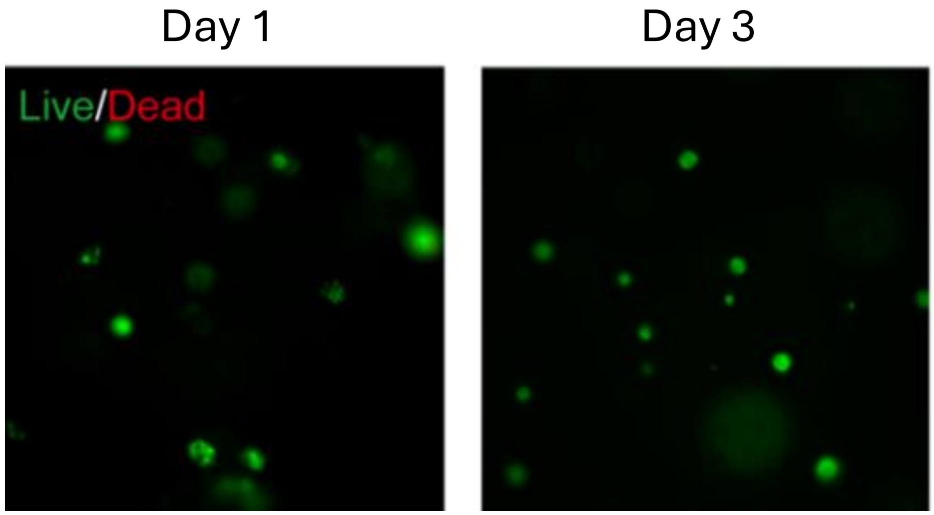

Supplementary Figure 12. Live/Dead staining images of 3D LC-hiPSC colonies after DLP bioprinting on day 1 and 3.

2. Additional Experimental Section

2.1. Methods for hiPSC lumen structure quantification:

3D hiPSC colonies harvested from PGmatrix were stained with Acridine Orange and photographed with Zeiss Axio Vert A1 Microscope. Images obtained were processed and total number of 3D colonies was counted with Fiji-imageJ, 3D colonies with lumen structure showed a dark center with clear green bright edge, those without lumen structure were manually counted and subtracted to obtain the number of 3D colonies with lumen structure. Ratio of 3D hiPSC colonies with lumen = number of 3D colonies with lumen/ total number of 3D hiPSC colonies x 100%. Four images with over 500 3D colonies were used for quantification.

2.2 Tri-lineage differentiation of live shipped hiPSCs

Luminal cyst hiPSC (LC-hiPSC) 3D colonies were retrieved from 3D culture and were immediately encapsulated in PepGel hybrid PGmatrix-LiveShip hydrogel (PepGel LLC) in a conical centrifuge tube following manufacturer’s instructions and shipped overnight to Applied

Stemcell Inc in California under ambient condition. Upon receipt of the live shipped LC-hiPSCs, the cells were retrieved from PGmatrix-LiveShip hydrogel by diluting with DPBS followed by centrifugation. The recovered LC-hiPSCs were characterized following traditional 2D methods in order to compare with local hiPSCs cultured from 2D. Therefore, the recovered LC-hiPSCs plated into Matrigel (Corning) coated 6-well plate and expanded with mTeSR1 medium (Stemcell Technologies). After reaching appropriate confluency, LC-hiPSCs were then treated with Knockout DMEM supplemented with Activin and Wnt3 for Endoderm induction, with BMP4 and Activin for Mesoderm induction, or with Dorsomorphin, SB431542 and Noggin for Ectoderm induction. After induction, cells were fixed and stained for Brachyury and GATA4 (mesoderm), Pax6 and Tuj1 (ectoderm), Sox17 and Fox2A (endoderm).

2.3 Bioink pre-screening

Materials: PepGel hybrid (PGmatrix Spheroids (PGS) kit) was provided by PepGel LLC. Polyethylene Glycol Diacrylate (PEGDA) with 6000 MW was purchased from Advanced Biomatrix (#5339). Photoinitiator lithium phenyl-2,4,6-trimethylbenzoylphosphonate (LAP) was from Sigma-Aldrich.

Bioink pre-screening of UV curing: The bioink solution was plated in a mold by pipetting at 50 µl/disk to yield a disk with dimension of 8 mm × 1 mm (diameter × thickness). Each formula had three replicates. Dymax BlueWave MX-150 (Dymax Corporation, Torrington, CT, USA) was used to crosslink the hydrogel under UV irradiation at density of 80 mW/cm^2 for 35 seconds, and wavelength of 405 nm. The UV intensity was calibrated by a radiometer Accu-Cal 50-L (Dymax) every time before the experiment.

Bioink pre-screening of mechanical test: Dynamic mechanical analysis (DMA) was conducted using a TA Q800 DMA analyzer (TA Instruments, New Castle, DE, USA) under compressive mode at an oscillation frequency of 1 Hz and oscillation strain of 1% (linear viscoelastic region). The hydrogel specimens (8 mm × 1 mm) from the mold were placed on the lower parallel plate, after which the upper parallel plate was lowered until contact with the surface of the hydrogel was made. To ensure full contact of the plate on the sample, a preload force of 0.05 N was used. All the tests were performed in an ambient environment (25 °C). Compressive storage modulus G' was obtained to represent the hydrogel strength.

hiPSC cell encapsulation and 3D cell culture: hiPSC 3D colonies harvested from 3D culture described above were used for bioink cell encapsulation and bioprinting. Spheroid pallets were suspended in the hydrogel bioink solution (**Supplementary Table 5**) at a seeding density of 200,000 3D colonies/ml. The mixture was printed to the PLA mold (50 µl/disk) by pipetting and ready for UV irradiation. The cured hydrogel was removed from mold and immersed to 1 ml of mTeSR media supplemented with ROCK inhibitor Y27632 (Stemcell Technologies). To feed cells, 1 ml of fresh media was replaced daily until day 3.

Evaluations of mechanical properties: A 6800 SERIES mechanical tester (Instron, Norwood, MA, USA) was used to measure the mechanical properties of constructs, with data analysis performed using Bluehill Universal (v4.13, Instron) and origin (v9.0, OriginLab, Northampton, MA, USA). For tensile testing, specimens with dimensions of 3 cm in length, 1 cm in width, and 3 mm in height were utilized, while for compression testing, specimens with dimensions of 1 cm in length, 1 cm in width, and 5 mm in height were employed.

Supplementary Table 1. Differentiation media formulation

Embryoid Body (EB) Medium			
Reagent	Ratio (%)	Supplier	
DMEM/F12	93	Thermo Fisher	
Embryonic stem-cell qualified fetal bovine serum (ES-FBS)	5	Thermo Fisher	
Insulin-Transferrin-Selenium-Sodium Pyruvate (ITS-A) (100x)	1	Thermo Fisher	
55mM 1-Thioglycerol	0.1	Sigma-Aldrich	
Antibiotic-Antimycotic solution	1	Thermo Fisher	
Differentiation Supplements			
Day	Reagent	Final conc. (ng/ml)	Stock conc. (μg/ml)
0<br>2	FGF-2/bFGF	10	100
	Activin A	100	100
	TGFβ	10	100
4	FGF-4	10	100
6	BMP-4	10	100
8	WIF-1	1000	200
10	DKK-1	100	100
12	HGF	50	100
14	OSM	30	100

Supplementary Table 2. Gene and primers for RT-qPCR

**Gene**		**Sequence**	**T_m**
ALB	Forward	GATCTGCTTGAATGTGCTGATG	60
	Reverse	ATGCAGTGGGATTTTTCCAA	

HNF4A	Forward	GTGGACAAAGACAAGAGGAACC	60
	Reverse	GCTGTCCTCATAGCTTGACCTT	
A1AT	Forward	GGTGCTGCTGATGAAATACCTG	60
	Reverse	TTTTCCAGGTGCTGTAGTTTCC	
AFP	Forward	CTGCACTTCTTCATATGCCAAC	60
	Reverse	GAGGGACATATGTTTCATCCAC	
CYP3A7	Forward	TGACTAGAGCAAGTTTCATGTTC AC	60
	Reverse	CCTCCCTGAAAGGTTCAGTAAA	
ASGR1	Forward	GAGACGTTCAGCAACTTCACAG	60
	Reverse	CTCAGGTCCTTCTGCTGTTTCT	
CYP3A4	Forward	GTATGGAAAAGTGTGGGGCTTT	60
	Reverse	CATTCTTTCACTAGCACTGTTTTG	
TDO2	Forward	GCAAAAAGAGGTGCTACTGTCC	60
	Reverse	CTCCCTGAAGTGCTCTGTATGA	
TTR	Forward	AATCCAAGTGTCCTCTGATGGT	60
	Reverse	GCTCTCCAGACTCACTGGTTTT	
PSMB6[1]	Forward	GAAGACCTGATGGCGGGA	60
	Reverse	CGGAGCCTCCAATGGC	
OCT4	Forward	AAAGAGAAAGCGAACCAG	52
	Reverse	CCACATCCTTCTCGAGCC	
SOX2	Forward	CAACCAGAAAAACAGCCC	52
	Reverse	TCTCCGACAAAAGTTTCC	
SOX17[2]	Forward	CGCACGGAATTTGAACAGTA	60
	Reverse	GGATCAGGGACCTGTCACAC	
FOXA2[3]	Forward	AGATGGAAGGGCACGAGC	63
	Reverse	CAGGCCGGCGTTCATGTT	
CDX2[2]	Forward	GGGCTCTCTGAGAGGCAGGT	63
	Reverse	CCTTTGCTCTGCGGTTCTG	
CEBPA[2]	Forward	AGAAGTCGGTGGACAAGAACAG CA	66
	Reverse	ATTGTCACTGGTCAGCTCCAGCA	

PROX1[2]	Forward	GGGCTCTCCTTGTCGCTCATAAA	65
	Reverse	GGTAATGCATCTGTTGAACTTTA CGTC	
TBX3[2]	Forward	TTACCAAGTCGGGAAGGCGAAT	66
	Reverse	CATCCTCTTTGGCATTTCGGGG	
CAPN10	Forward	GGAGGTGACCACAGATGACC	60
	Reverse	GTAAGGGGAGCCAGAACACA	
EID2	Forward	GAAGCCTGCAGAGCAAGG	60
	Reverse	ATATCGAGGTCCACCCTGTG	
ZNF324B	Forward	GAGAATGGCCACGAGCTTT	60
	Reverse	TTTACACTGTGGCAGGCATC	

Supplementary Table 3. Antibodies for Immunofluorescence

Antibody	Supplier	Dilution Ratio
Phalloidin-iFluor 488	Abcam	1:1000
alpha Tubulin Monoclonal Antibody (DM1A)	Thermo Fisher	1:400
Rabbit AFP Polyclonal Antibody	Thermo Fisher	1:200
Rabbit Albumin Polyclonal Antibody	Thermo Fisher	1:200
Mouse HNF4A Monoclonal Antibody (K9218)	Thermo Fisher	1:1000
Rabbit ABCB11 Polyclonal Antibody	Thermo Fisher	1:500
Mouse Anti-alpha 1 Sodium Potassium ATPase antibody	Abcam	1:500
Goat anti-Rabbit IgG (H+L) Cross-Adsorbed Secondary Antibody, Alexa Fluor™ 488	Thermo Fisher	1:500
Goat anti-Mouse IgG (H+L) Highly Cross-Adsorbed Secondary Antibody, Alexa Fluor™ Plus 647	Thermo Fisher	1:500

Supplementary Table 4. Antibodies for Immunohistochemistry

Antibody	Supplier	Dilution Ratio
Hepatocyte paraffin 1 (OCH1E50) Mouse antibody	Dako Corporation	1:50
E-cadherin (4A2) Mouse monoclonal antibody	Cell Signaling Technology	1:100
Ki-67 (MIB-1) Rabbit monoclonal antibody	Biocare Medical	N/A
Rabbit Albumin Polyclonal Antibody	Leica	N/A
Cytokeratin 19 (CK19) (b170) Mouse Monoclonal Antibody	Thermo Fisher	1:1000
Claudin-5 (4C3C2) Mouse monoclonal antibody	Invitrogen	1:90
Antibody for *in vivo* samples		
rabbit monoclonal anti-hepatocyte specific antigen	Sigma	1:200
rabbit monoclonal anti-ALDH1A1	Cell Signaling Technology	1:200

Supplementary Table 5. Bioink solution formulation at different PEGDA and PGS ratios

Bioink solution (1 mL)	PEGDA stock solution	PGS solution	LAP stock solution	Cell media w/wo hiPSCs
1.5% PEGDA + 3% PGS, 0.2% LAP	75 µL	750 µL	66.7 µL	108.3 µL
3% PEGDA 0.2% LAP	150 µL	--	66.7 µL	783.3 µL
3% PEGDA + 3% PGS, 0.2%LAP	150 µL	750 µL	66.7 µL	33.3 µL
5% PEGDA + 2% PGS, 0.2% LAP	250 µL	500 µL	66.7 µL	183.3 µL
5% PEGDA 0.2% LAP	250 µL	--	66.7 µL	683.3 µL

Supplementary Table 6. LC-hiPSC–derived hepatic organoids (PG-hiHs) cultured in PGmatrix suspension (PG-sus) exhibited significantly higher differentiation efficiency than hepatic aggregates cultured in U96 wells.

Sample	Albumin (%)	HNF4A (%)
Control hiPSC	26.30±3.2[c]	0.00±0.00[c]
Hepatic aggregate in U96	77.80±6.3[b]	30.8±2.6[b]
PG-hiH in 3D PG-sus	94.8±3.9[a]	39.6±3.1[a]

References

1. An extended ΔCT-method facilitating normalisation with multiple reference genes suited for quantitative RT-PCR analyses of human hepatocyte-like cells - PubMed. https://pubmed.ncbi.nlm.nih.gov/24658132/.
2. Ang, L. T. *et al.* A Roadmap for Human Liver Differentiation from Pluripotent Stem Cells. *Cell Rep.* **22**, 2190–2205 (2018).
3. Hay, D. C. *et al.* Efficient differentiation of hepatocytes from human embryonic stem cells exhibiting markers recapitulating liver development in vivo. *Stem Cells Dayt. Ohio* **26**, 894–902 (2008).
4. 22. Li, Q. et al. Universal Peptide Hydrogel for Scalable Physiological Formation and Bioprinting of 3D Spheroids from Human Induced Pluripotent Stem Cells. Adv. Funct. Mater. 31, 2104046 (2021).
5. Pettinato, G. et al. Scalable Differentiation of Human iPSCs in a Multicellular Spheroid-based 3D Culture into Hepatocyte-like Cells through Direct Wnt/β-catenin Pathway Inhibition. Sci. Rep. 6, 32888 (2016)
